# 3D Conjugated Nonflat Biphenyl Side Chains: Their Exclusive Role in Inducing Negative Electrostatic Potential in Efficient Organic Solar Cells

**DOI:** 10.1002/smll.202509667

**Published:** 2025-08-28

**Authors:** Seonghun Jeong, Zhe Sun, Yongjoon Cho, Sangjin Yang, Thi Le Huyen Mai, Changduk Yang

**Affiliations:** ^1^ School of Energy and Chemical Engineering Ulsan National Institute of Science and Technology (UNIST) 50 UNIST‐gil, Ulju‐gun Ulsan 44919 South Korea; ^2^ Graduate School of Carbon Neutrality Ulsan National Institute of Science and Technology (UNIST) 50 UNIST‐gil, Ulju‐gun Ulsan 44919 South Korea

**Keywords:** biphenyl side chain, electrostatic potential, nonfullerene acceptor, nonradiative energy loss, organic solar cell

## Abstract

In nonfullerene acceptor (NFA) (so‐called Y‐series)‐based organic solar cells (OSCs), reducing energy loss, particularly nonradiative energy loss, is critical for achieving high power conversion efficiency (PCE). However, at present, molecular design strategies for controlling and/or optimizing energy loss involve multiple complexities. Therefore, this study demonstrates that the introduction of simple, 3D conjugated nonflat biphenyl side chains into a Y‐series NFA (yielding BPY) can improve high dipole moments‐induced intermolecular interactions and negatively charged surface electrostatic potential driven by partially isolated negative charges. When BPY is blended with a PM6 donor, the resulting BPY‐based OSC exceeds that of the control Y6‐based OSC. This result is attributed to the minimized nonradiative energy loss via weakened π–π stacking and efficient charge dynamics. The addition of BPY to PM6:BTP‐eC9 blends yields ternary OSCs that exhibit a high PCE of 19.23% owing to significant improvements in both the open‐circuit voltage and fill factor. These findings not only highlight the influence of biphenyl side chains on the molecular properties of NFAs but also provide an effective material avenue to optimize nonradiative energy loss for high‐performance OSCs.

## Graphical abstract

A nonfullerene acceptor named BPY, featuring 3D biphenyl side chains, is developed to reduce nonradiative energy loss in organic solar cells. The improved dipole moments, negatively charged surface electrostatic potential, and proper morphology of BPY enhance charge dynamics and reduce nonradiative recombination, enabling high‐performance OSCs with PM6:BTP‐eC9 to reach 19.23% power conversion efficiency.

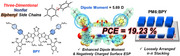

## 1 Introduction

Organic solar cells (OSCs) are one of the promising next‐generation energy harvesting platforms because of their unique advantages, including lightweight, flexibility, transparency, and solution processability.^[^ ^1^, ^2^, ^3^ ^]^ To date, as part of the numerous efforts to improve the power conversion efficiency (PCE) of OSCs, the emergence of novel A–DA'D–A‐type nonfullerene acceptors (NFAs), so‐called Y‐series NFAs, provides an avenue to fabricate single‐junction and ternary OSCs with PCEs exceeding 19%.^[^ ^4^, ^5^, ^6^, ^7^, ^8^, ^9^ ^]^ Such Y‐series NFAs possess notable features such as high light harvesting ability and charge mobility with their superior morphological features, leading to a significant enhancement in short‐circuit current density (*J*_SC_), fill factor (FF), and PCE.^[^ ^10^, ^11^, ^12^ ^]^ Therefore, many researchers have focused on further modulating the crystalline features and intermolecular packing of Y‐series NFAs to optimize their *J*_SC_ and FF values using molecular engineering.^[^ ^13^, ^14^, ^15^, ^16^ ^]^ However, the debate regarding open‐circuit voltage (*V*_OC_) improvement‐induced PCE increases remains relatively limited and complex because enhancing *V*_OC_ requires a delicate molecular approach that considers the trade‐off between *V*_OC_ and *J*_SC_, maintenance of absorption behaviors, and improving morphological characteristics.

The main strategies for increasing the *V*_OC_ of OSCs are tuning the frontier molecular orbitals (FMOs)/bandgap of active layer materials and minimizing the energy loss (*E*_loss_), which is defined as Δ*E*_1_ (radiative recombination above the bandgap) + Δ*E*_2_ (radiative recombination below the bandgap) + Δ*E*_3_ (nonradiative recombination).^[^ ^17^ ^]^ Among these two strategies, reducing nonradiative energy loss is a more facile and efficient approach to improve *V*_OC_ because changes in the absorption and bandgap of NFAs are prevented.

To minimize nonradiative energy loss in OSCs, there are relevant criteria in terms of the molecular engineering of Y‐series NFAs: i) higher dipole moment, ii) proper electronic structures, iii) good compatibility with donor materials, and iv) favorable surface morphology with lower defect/trap density.^[^ ^18^, ^19^, ^20^, ^21^, ^22^ ^]^ In this context, the design of asymmetric molecules is effective in reducing nonradiative energy loss in bulk heterojunctions by increasing the molecular dipole moment, tuning the electronic structures, and facilitating favorable active layer morphologies.^[^ ^19^ ^]^ However, this approach significantly increases the synthesis cost and complexity. Another effective strategy is the incorporation of bulky side chains, which can optimize crystalline features and surface morphologies, leading to minimized nonradiative energy loss in OSCs.^[^ ^10^ ^]^ However, this strategy is insufficient for modulating the dipole moment and electronic structure of single molecules, leaving room for further modification to minimize nonradiative energy loss.

From this point of view, we expect that the introduction of 3D biphenyl side chains into Y‐series NFAs can be a useful and effective method for reducing nonradiative energy loss in OSCs while integrating the aforementioned criteria. This approach holds promise for achieving enhanced dipole moments and morphological advantages without compromising spectral compatibility and energetic alignment with the donor polymer.

In this work, we synthesized a biphenyl side chain‐incorporating Y‐series NFA, BPY. Through comprehensive optical characterization, we revealed that 3D conjugated nonflat biphenyl side chains enhance the aggregation behavior and polar excited state of BPY. The results of computational simulation demonstrate that BPY exhibits a dramatically enhanced dipole moment and a properly controlled electronic structure. In addition, BPY‐based films exhibit fibrillar nanoscale morphologies and properly rearranged crystalline behavior, i.e., suppressed π–π stacking. Consequently, the BPY‐based OSC achieved a superior *V*_OC_ value of 0.883 V and a PCE of 18.06% owing to the minimized nonradiative energy loss (0.201 eV) compared to the Y6‐based OSC (0.238 eV). Furthermore, the incorporation of BPY into the PM6:BTP‐eC9‐based OSC increased the PCE up to 19.23%, accompanied by increases in both *V*_OC_ and FF. These results provide a molecular engineering strategy for fabricating high‐performance OSCs based on Y‐series NFAs by minimizing nonradiative energy loss.

## 2 Results and Discussion

### 2.1 Material Synthesis and Characterization

**Figure 1:**
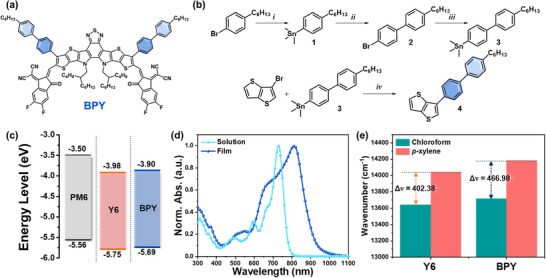
a) Chemical structure of BPY and b) synthetic route for 3‐(4′‐hexyl‐[1,1′‐biphenyl]‐4‐yl)thieno[3,2‐*b*]thiophene core unit. i) *n*‐BuLi, Sn(Me)_3_Cl, THF, ‒78 °C, ii) 1,4‐dibromobenzene, Pd(PPh_3_)_4_, Tol, 110 °C, iii) *n*‐BuLi, Sn(Me)_3_Cl, THF, ‒78 °C, and iv) Pd(PPh_3_)_4_, Tol, 110 °C. c) Illustration of energy levels of PM6, Y6, and BPY. d) UV–vis absorption spectra of BPY in chloroform solution and corresponding thin‐film state. e) Wavenumber for maximum absorption points for Y6 and BPY in chloroform and *p*‐xylene solutions.

The chemical structure and synthesis routes of BPY are illustrated in **Figure** **1**a and Scheme 1 (see Experimental Section, Scheme S1, and Figures S21–S27, Supporting Information, for the detailed procedures and characterizations). The incorporation of outer biphenyl side chains into a Y‐series NFA was achieved using repetitive synthesis protocols (e.g., stannylation and palladium‐catalyzed Stille cross‐coupling reactions using aryl bromides and 3‐bromothienothiophene (Figure 1b). 3‐(4′‐Hexyl‐[1,1′‐biphenyl]‐4‐yl)thieno[3,2‐*b*]thiophene (compound 4) underwent a sequence of common reactions involved in the synthesis of Y‐series NFAs (e.g., stannylation, Stille coupling, Cadogan reductive cyclization, Vilsmeier–Haack formylation, and Knoevenagel condensation) to afford the target BPY.^[^ ^12^, ^23^, ^24^ ^]^ BPY exhibits good solubility in common OSC‐processing solvents, such as chloroform (CF), chlorobenzene, and xylenes, at room temperature, which can facilitate its solution processing during device fabrication. In thermogravimetric analysis and differential scanning calorimetry measurements, BPY exhibited higher thermal stability than Y6, with 5% weight‐loss decomposition and melting temperatures of 346.2 and 332.4 °C, respectively (Figures S1 and S2, Supporting Information).

### 2.2 Electrochemical and Optical Properties

Cyclic voltammetry (CV) was performed to evaluate the FMO energy levels of NFAs, where the archetypal Y6 NFA was selected for comparative analysis in the following sections. Y6 and BPY exhibited the highest occupied molecular orbital (HOMO)/lowest unoccupied molecular orbital (LUMO) levels of –5.75/–3.98 and –5.69/–3.90 eV, respectively (Figure 1c and Figure S3, Supporting Information and **Table** **1**). Compared with Y6, the higher FMO energy levels of BPY are attributed to the electron‐donating properties of biphenyl side chains, which can increase *V*_OC_ (due to higher LUMO energy levels).^[^ ^25^ ^]^

**Table 1:** Optical and electrochemical properties of Y6 and BPY.

	*λ*_sol_^max^ [nm]^a)^	*λ*_film_^max^ [nm]^a)^	*λ*_onset_ [nm]^b)^	*E*_g_^opt^ [eV]^b)^	*E*_HOMO_^CV^ [eV]^c)^	*E*_LUMO_^CV^ [eV]^c)^
Y6	729	829	915	1.36	−5.75	−3.98
BPY	729	812	918	1.35	−5.69	−3.90

^a)^ Taken from the material solutions in xylenes and corresponding films on glass substrates;

^b)^ Determined from the onset of the UV‐vis absorption plots in the films;

^c)^ Estimated from the oxidation and reduction onset by using the equations of *E*_HOMO_ = −(*E* ^onset^_ox_ − *E* ^onset^_Fc_ + 4.8) eV, *E*_LUMO_ = −(*E* ^onset^_red_ − *E* ^onset^_Fc_ + 4.8) eV, and *E* ^CV^_g_ = *E*_HOMO_ − *E*_LUMO._

Ultraviolet–visible (UV–vis) absorption spectroscopy was used to elucidate the optical properties of Y6 and BPY in CF solution and film states. As shown in Figure 1d, in the CF solution, both Y6 and BPY exhibited strong absorption in the range of 500–800 nm, with an identical maximum absorption peak (*λ*_max_) located at 729 nm and a similar absorption coefficient (≈1.35 ×10^5^ M^−1^ cm^−1^) (Figure S4, Supporting Information). In the films, BPY exhibited a bathochromic shift of 83 nm, resulting in *λ*_max_ at 812 nm, which is shorter than that of Y6 (*λ*_max_ = 829 nm). Furthermore, the BPY film exhibited a more pronounced vibronic shoulder peak than the Y6 film, ranging from 600 to 750 nm, owing to extended conjugation by hexylbiphenyl side chains.^[^ ^25^ ^]^ The optical bandgaps (E_g_^opt^s) of Y6 and BPY are 1.36 and 1.35 eV, respectively, as calculated by the absorption onset (*λ*_onset_) points.

The degree of the solvatochromic shift was estimated by comparing the *λ*_max_ values of Y6 and BPY in solutions containing polar CF and less‐polar *p*‐xylene (Figure 1e and Figures S5 and S6, Supporting Information). Interestingly, BPY exhibited a higher degree of solvatochromic shift of 466.98 cm^−1^ than Y6 (402.38 cm^−1^), indicating that BPY forms a more polar excited state because of its higher dipole moment.^[^ ^26^, ^27^ ^]^ By lowering the Coulomb binding energy of excitons, BPY can improve exciton dissociation at the junction between the active layer and donor materials.^[^ ^27^, ^28^ ^]^

### 2.3 Computational Simulation

Density functional theory calculations were performed using the B3LYP/6‐31G(d) level to demonstrate the effect of the hexylbiphenyl side chains. As shown in Figures S7 and S8 (Supporting Information), the LUMOs were delocalized across all molecules, whereas the HOMOs were primarily centralized on the central DA'D core frameworks in Y6 and BPY. The calculated HOMO/LUMO energy levels of Y6 and BPY are –5.77/–3.74 and –5.68/–3.64 eV, respectively, which agree well with the CV data and the above discussion.

**Figure 2:**
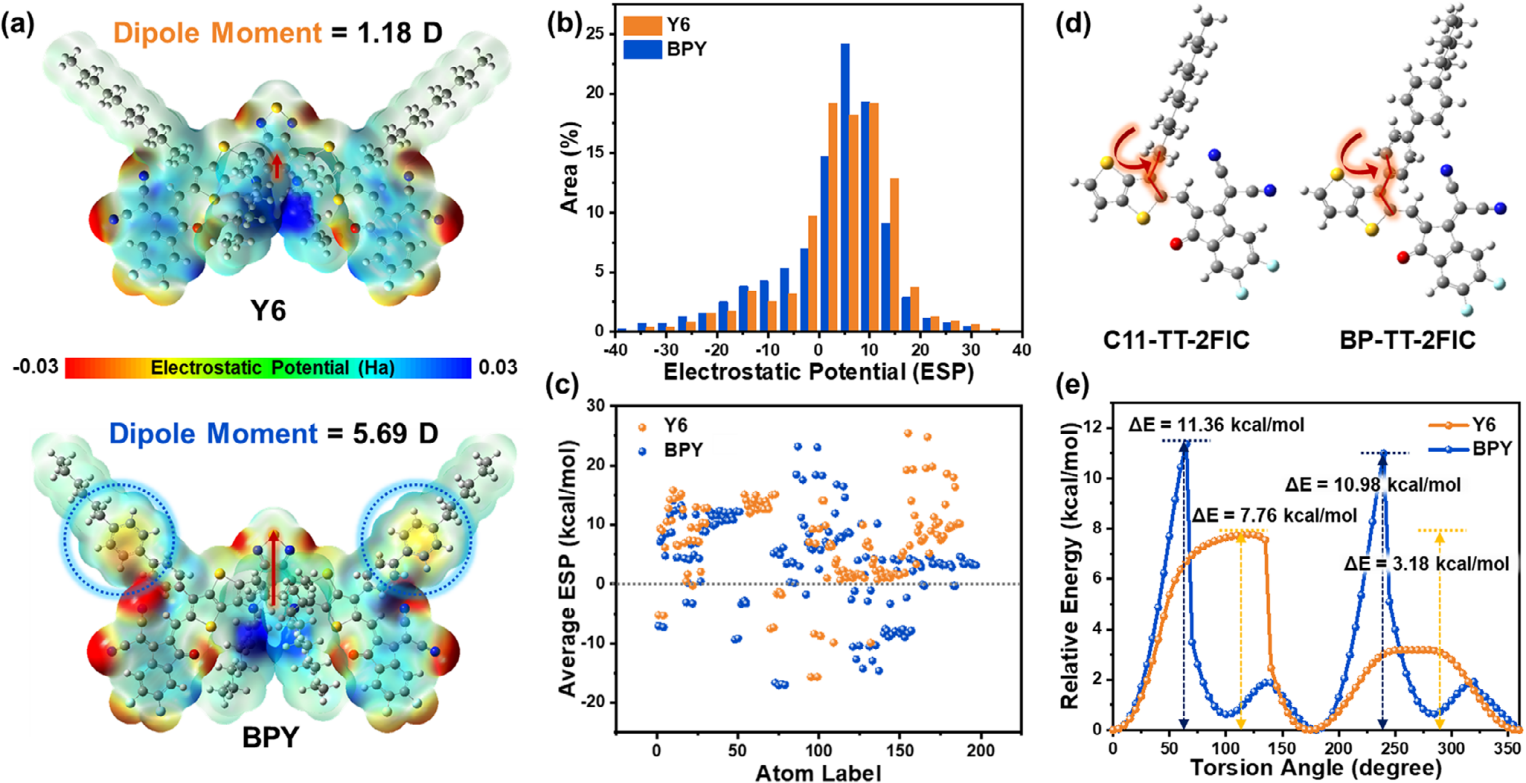
a) Electrostatic potential (ESP) mapping and dipole moment (red arrows) of Y6 and BPY. b) Statistical ESP area distribution and c) average ESP values of each atoms. d) Subunits of Y6 and BPY and e) relaxed potential energy scans of subunits.

Notably, BPY exhibits a dipole moment value of 5.69 Debye (D), which is approximately five times that of Y6 (1.18 D) (**Figure** **2**a), suggesting that BPY molecules exhibit stronger electrostatic interactions.^[^ ^20^ ^]^ To further explore the dipole moment variations, we calculated the local dipole moments of the outer side groups alone. As shown in Figure S9 (Supporting Information), the hexylbiphenyl side group affords a dipole moment value of 0.5278 D, which is higher than those of the *n*‐undecyl (0.0421 D) and phenylhexyl (0.3887 D) groups.

Furthermore, we investigated the electrostatic potential (ESP) on the van der Waals surface, with the isosurface set at 0.001 a.u. for Y6 and BPY. We found that the surface regions in the conjugated backbone of both NFAs are positively charged, accompanied by partially negatively charged distributions at cyano (C≡N) and carbonyl (C═O) functionalities in the terminal groups and imines (C = N) in the central thiazole unit. Because of their nonflat conformation, the second phenyl rings within the outer biphenyl side groups of BPY exhibit partially isolated negative charges, possibly creating additional electrostatic interaction points.^[^ ^1^ ^]^ The quantified ESP area distribution and average ESP (ESP_avg_) values corresponding to single atoms of Y6 and BPY were estimated using the Multiwfn program.^[^ ^29^ ^]^ The relevant data are summarized in **Table** **2**.

**Table 2:** Ratio of negative ESP surface area, molecular surface area, MPI, extreme value of ESP, and total average ESP of Y6 and BPY (isosurface = 0.001 au).

	Overall surface area [Å^2^]	Negative ESP surface area [%]	MPI [kcal mol^−1^]	Minimal value [kcal mol^−1^]	Maximal value [kcal mol^−1^]	Overall average value [kcal mol^−1^]
Y6	1461.94	23.73	8.75	−35.60	34.02	4.45
BPY	1754.80	24.81	9.11	−37.43	32.11	3.06

As shown in Figure 2b,c, the ESP area distribution of BPY shifted in the negative direction, along with a higher negative ESP surface area of 24.81% than that of Y6 (23.73%), resulting in a decrease in its overall ESP_avg_ value (3.06 kcal mol^−1^ for BPY and 4.45 kcal mol^−1^ for Y6). This is probably due to the contribution of the negatively charged phenyl rings in the hexylbiphenyl side chains to the electronic structure of the overall molecular region. Considering that most donor polymers exhibit negative ESP_avg_ values, in terms of the ESP difference (ΔESP_avg_), the less positive overall ESP_avg_ value of BPY can induce smaller ΔESP_avg_ values with donor polymers, which can result in reduced donor−acceptor compatibility.^[^ ^22^, ^30^ ^]^ Nevertheless, BPY exhibits a higher molecular polarity index value of 9.11 kcal mol^−1^ than Y6 (8.75 kcal mol^−1^), indicating its higher polarity associated with its larger dipole moment, which can positively affect the construction of well‐intermixed blend morphology and promote efficient exciton dissociation in the active layer.^[^ ^31^ ^]^

In addition, we conducted relaxed potential energy scans for the conformer building segments of 3‐alkylthienothiophene–2FIC (defined as C11‐TT‐2FIC for Y6 and BP‐TT‐2FIC for BPY) as the rotation of the side chains (Figure 2d,e). For both NFAs, optimized conformations were detected at 180° with a relative energy of zero. In the case of the BP‐TT‐2FIC conformer, multiple low‐energy states with relative energies of 0.62, 0.66, and 0.01 kcal mol^−1^ at 100°, 280°, and 360° (= 0°), respectively, and notable double transition‐state relative energies of 11.36 and 10.98 kcal mol^−1^ at 65° and 240°, respectively, were also observed. In contrast, the C11‐TT‐2FIC conformer possessed a single low‐energy state with a relative energy of approximately zero at 360° (= 0°) and double transition‐state relative energies of 7.76 and 3.18 kcal mol^−1^ at 115° and 275°, respectively. Based on these results, the BP‐TT‐2FIC conformer tends to adopt restricted rotation and maintain its energetically stable conformation compared to the C11‐TT‐2FIC conformer, because of the larger steric hindrance of the bulky biphenyl moiety.^[^ ^32^ ^]^

### 2.4 Photovoltaic Performance and Charge Dynamics

**Figure 3:**
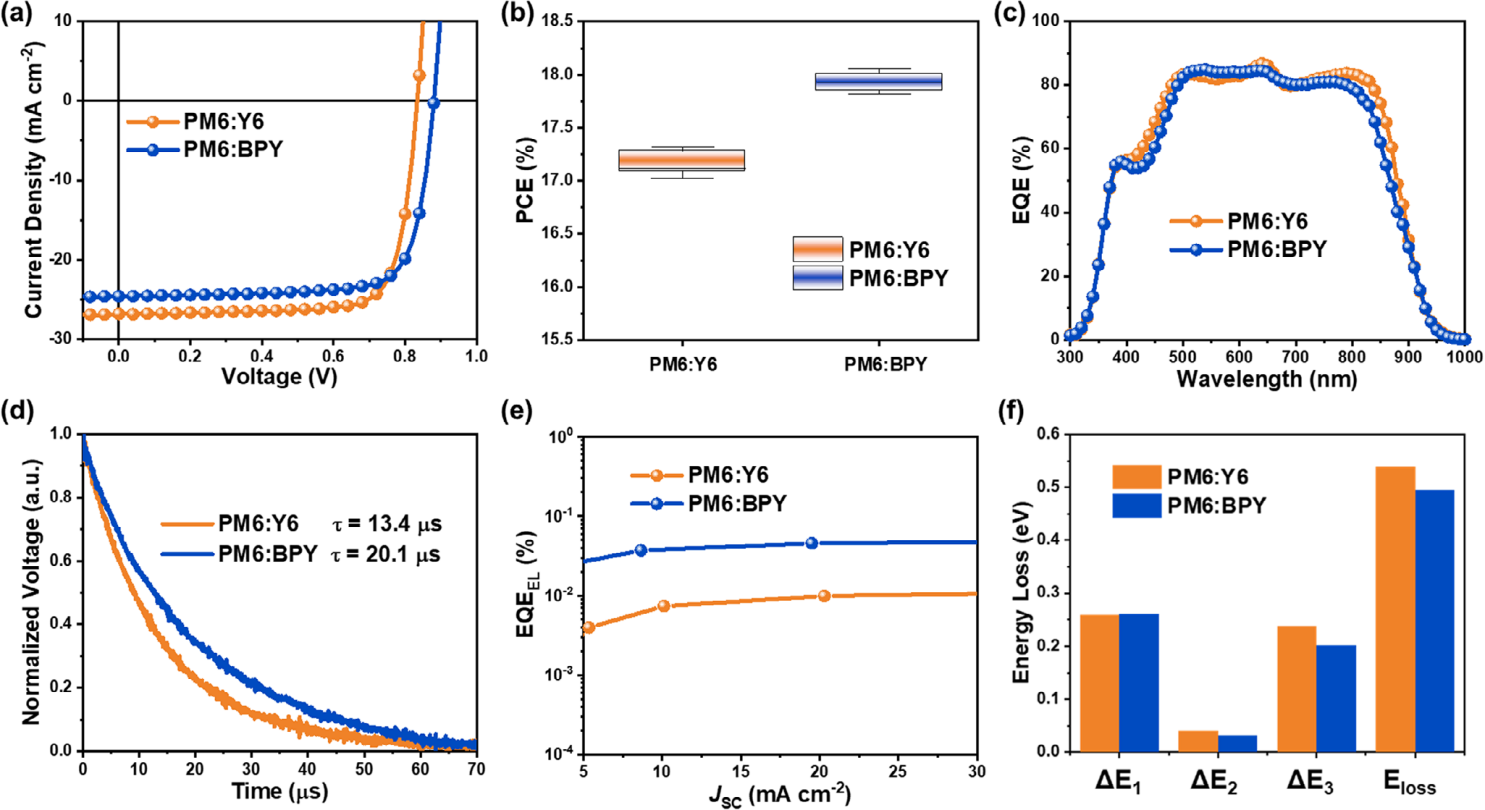
a) *J*–*V* curves and b) statistical PCE distribution, and c) EQE spectra of optimized OSCs based on PM6:Y6 and PM6:BPY. d) TPV plot obtained from optimized OSCs employing Y6 and BPY. e) EQE_EL_ and f) energy losses of Y6‐ and BPY‐based OSCs.

We selected PM6 as the donor material for the active layer and fabricated OSCs using the conventional ITO/PEDOT:PSS/PM6:NFA/H75/Ag architecture to investigate the effect of incorporating hexylbiphenyl side chains into acceptor structures on OSC performance. The optimized current density–voltage (*J−V*) characteristics and statistical distribution of the PCEs of the OSCs are presented in **Figure** **3**a,b, respectively, with the parameters detailed in **Table** **3**. The Y6‐based OSC achieved a PCE of 17.32%, *J*_SC_ of 27.03 mA cm^−2^, *V*_OC_ of 0.832 V, and FF of 76.44%. In contrast, the BPY‐based OSC exhibited higher *V*_OC_ and FF values of 0.883 V and 78.32%, respectively, but a lower *J*_SC_ value of 26.15 mA cm^−2^, resulting in an improved PCE of 18.06%. In addition, we used BPY as the secondary acceptor in the PM6:BTP‐eC9 host system to fabricate ternary OSCs, achieving a remarkable PCE of 19.23%, along with concurrent improvements in both *V*_OC_ and FF (**Table** **4** and Figure S10, Supporting Information). Furthermore, we carried out a 200 h thermal stability test at 65 °C for Y6‐ and BPY‐based OSCs. As shown in Figure S11 (Supporting Information), BPY‐based OSCs retained approximately 90% of their initial PCE after 200 h, whereas the Y6‐based ones retained about 85%. It indicates that introducing the biphenyl side chains into Y‐series NFA can enhance the thermal stability of the OSCs.

**Table 3:** Photovoltaic parameters of PM6:Y6 and PM6:BPY‐based OSCs under AM 1.5G illumination.

	*V*_OC_ ^a)^ [V]	*J*_SC_ ^a)^ [mA cm^−2^]	*J*_SC_^EQE^ ^b)^ [mA cm^−2^]	FF ^a)^ [%]	PCE ^a)^ [%]
PM6:Y6	0.832 (0.831 ± 0.002)	27.03 (26.82 ± 0.13)	26.31	76.44 (76.02 ± 0.46)	17.32 (17.13 ± 0.11)
PM6:BPY	0.883 (0.879 ± 0.004)	26.15 (26.01 ± 0.15)	25.43	78.23 (77.91 ± 0.45)	18.06 (17.88 ± 0.13)

^a)^ The average PCE values are obtained from 15 cells;

^b)^ Integrated from EQE spectra_._

**Table 4:** Photovoltaic parameters of PM6:BTP‐eC9 and PM6:BTP‐eC9:BPY‐based OSCs under AM 1.5G illumination.

	*V*_OC_ ^a)^ [V]	*J*_SC_ ^a)^ [mA cm^−2^]	*J*_SC_^EQE^ ^b)^ [mA cm^−2^]	FF^a)^ [%]	PCE^a)^ [%]
PM6:BTP‐eC9	0.842 (0.842 ± 0.003)	28.51 (28.20 ± 0.35)	27.82	77.31 (77.32 ± 1.13)	18.66 (18.35 ± 0.21)
PM6:BTP‐eC9:BPY	0.854 (0.853 ± 0.002)	28.31 (28.01 ± 0.36)	27.55	79.52 (78.68 ± 0.58)	19.23 (18.79 ± 0.25)

^a)^ The average PCE values are obtained from 12 cells;

^b)^ Integrated from EQE spectra.

The integrated *J*_SC_ values derived from the external quantum efficiency measurements were 26.31 and 25.43 mA cm^−2^ for the Y6‐ and BPY‐based devices, respectively, demonstrating good agreement with the *J−V* measurements (Figure 3c).

To investigate the charge recombination mechanisms, we analyzed the relationship between *J*_SC_ and light intensity (*P*_light_) (Figure S12, Supporting Information). This dependency follows the relation *J*_SC_ ∝ *P*_light_^α^, where α values approaching 1 indicate effective bimolecular recombination suppression. The calculated α values of the Y6‐ and BPY‐based devices were 0.98 and 0.99, respectively, suggesting comparable bimolecular recombination behavior.^[^ ^33^ ^]^ In addition, the relationship between *V*_OC_ and *P*_light_ yielded slopes of 1.22 and 1.30 *kT*/*q* for the Y6‐ and BPY‐based devices, respectively, indicating reduced trap‐assisted recombination in the BPY‐based device (Figure S13, Supporting Information).^[^ ^34^ ^]^ In addition, as shown in Figure 3d, transient photovoltage measurements were performed to investigate the charge recombination dynamics in both devices. The carrier lifetime (τ) in the PM6:BPY‐based device was significantly extended to 20.1 µs, compared to 13.4 µs in the PM6:Y6‐based device, indicating a lower charge recombination rate.^[^ ^34^, ^35^ ^]^ In the transient photocurrent measurement, both devices exhibited similar photocurrent decay times (0.456 and 0.473 µs for the PM6:Y6‐ and PM6:BPY‐based devices, respectively) (Figure S14, Supporting Information).

The charge transport properties of the blend films were evaluated using the space‐charge‐limited current method. As shown in Figure S15 (Supporting Information), the electron (*µ*_e_) and hole (*µ*_h_) mobilities for the Y6‐based device were 9.36 × 10^−4^ and 6.51 × 10^−4^ cm^2^ s^−1^ V^−1^, respectively, whereas the BPY‐based device exhibited *µ*_e_ and *µ*_h_ values of 7.53 × 10^−4^ and 6.37 × 10^−4^ cm^2^ s^−1^ V^−1^, respectively. The more balanced *µ*_e_/*µ*_h_ ratio (1.18) for the BPY‐based device (1.44 for the Y6‐based device) contributed to its enhanced FF. The exciton dissociation efficiency (*η*_diss_) and charge collection efficiency (*η*_coll_) were further examined through the relationship between the photocurrent density (*J*_ph_) and effective voltage (*V*_eff_) (Figure S16, Supporting Information). The BPY‐based device exhibited superior *η*_diss_ and *η*_coll_ values of 98.8% and 92.4%, respectively, compared to 97.3% and 90.2% of the Y6‐based device, reflecting improved charge dynamics and contributing to the higher FF of the BPY‐based device. The enhanced exciton dissociation probability of the BPY‐based device can be attributed to the higher dipole moment and lower Coulomb binding energy induced by the more polar excited state of BPY.^[^ ^28^, ^36^ ^]^

To comprehensively understand the improved *V*_OC_ of the BPY‐based device, we performed *E*_loss_ analysis (Figure 3f and **Table** **5**). The PM6:Y6‐ and PM6:BPY‐based devices exhibited Δ*E*_1_/Δ*E*_2_ values of 0.260/0.040 and 0.261/0.033 eV, respectively, indicating minimal differences in the radiative recombination losses. However, the BPY‐based device exhibited a significantly lower Δ*E*_3_ of 0.201 eV than 0.238 eV of the control Y6‐based device, which is attributed to the higher electroluminescence quantum efficiency (EQE_EL_) of the BPY‐based device (Figure 3e). This originates from the lower overall ESP value and weakened ESP‐induced intermolecular interactions between PM6 and BPY, which mitigate nonradiative recombination.^[^ ^18^ ^]^

**Table 5:** Detailed energy losses of the PM6:Y6‐ and PM6:BPY‐based devices.

Active layer	*E*_g_	*qV*_OC_	*qV*oc^SQ^	*qV*oc^rad^	△*E*_1_	△*E*_2_	△*E*_3_	*E*_loss_	EQE_EL_ (×10^−4^)
	[eV]	[eV]	[eV]	[eV]	[eV]	[eV]	[eV]	[eV]	
PM6:Y6	1.371	0.832	1.111	1.070	0.260	0.040	0.238	0.539	0.991
PM6:BPY	1.378	0.883	1.117	1.084	0.261	0.033	0.201	0.495	4.203

### 2.5 Film Morphology and Microstructure Analysis

**Figure 4:**
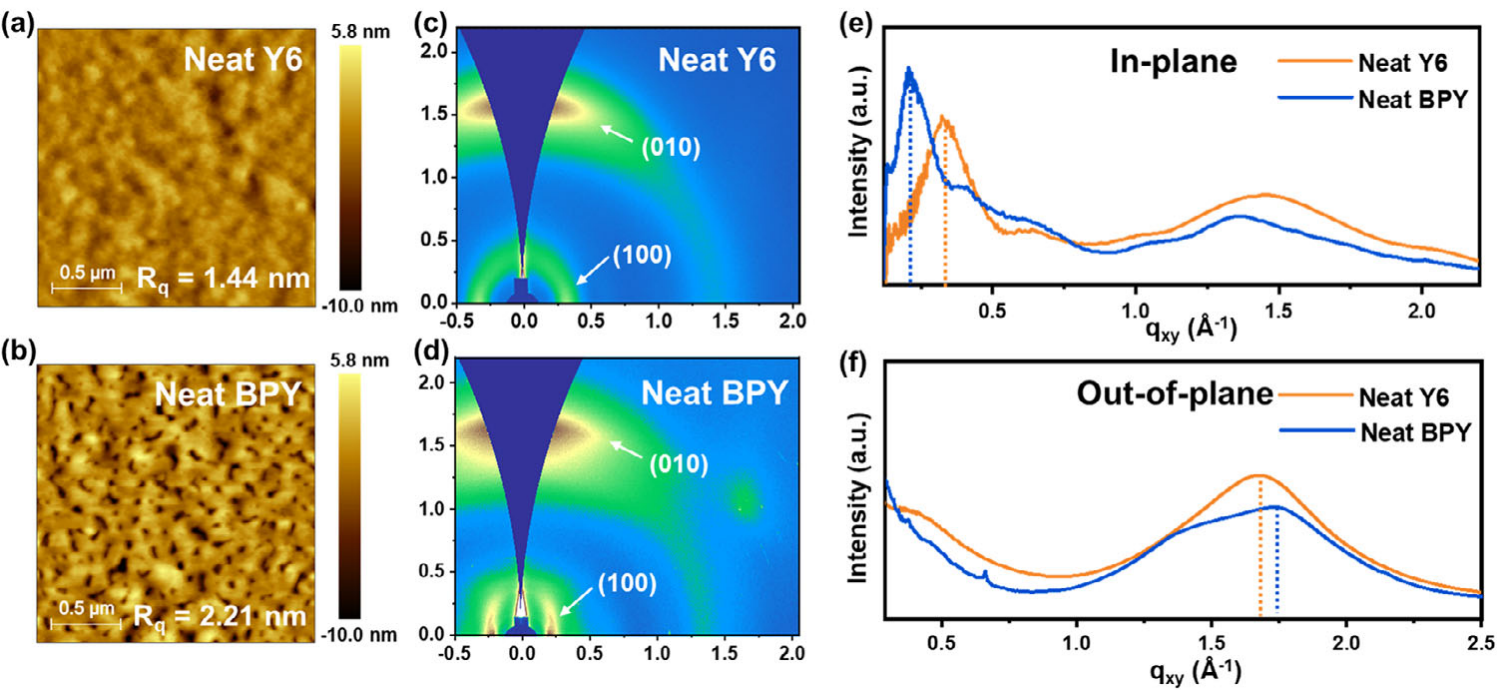
AFM height image of a) Y6 and b) BPY neat films. GIXD image of c) Y6 and d) BPY neat films and corresponding line‐cut graphs according to e) in‐plane and f) out‐of‐plane directions.

To gain a deeper understanding of the morphological characteristics of neat BPY and Y6 and their blend films with PM6, we performed atomic force microscopy (AFM) and grazing‐incidence X‐ray diffraction (GIXD) measurements. The experimental details are provided in the Experimental Section. As shown in **Figure** **4**a,b, the neat BPY film exhibited higher surface roughness (root‐mean‐square (*R*_q_) roughness of 2.21 nm) than the neat Y6 film (*R*_q_ = 1.44 nm). The neat BPY film exhibited a distinct hole‐like crater on its surface, which is attributed to enhanced molecular aggregation due to the higher dipole moment of BPY and electrostatic interactions induced by hexylbiphenyl side chains.^[^ ^37^ ^]^ The 2D GIXD patterns are presented in Figure 4c,d, and the corresponding 1D line‐cut profiles are provided in Figure 4e,f. The neat Y6 and BPY films exhibited a predominant face‐on orientation defined by obvious (010) π–π stacking peaks in the out‐of‐plane (OOP) direction and (100) lamellar stacking peaks in the in‐plane (IP) direction. Notably, the neat BPY film exhibited significantly larger *d*‐spacing and crystalline coherence length (CCL) values of 28.621 and 73.660 Å, respectively, than the neat Y6 film (*d*‐spacing = 18.802 Å and CCL = 50.698) in the IP direction (Table S1, Supporting Information). Despite the loose intermolecular packing induced by the nonflat hexylbiphenyl side chains of BPY, the similar numbers of lamellar stacks in Y6 and BPY (2.69 and 2.57, respectively) are probably attributed to the intensified intermolecular interactions governed by the modulated molecular dipole moment and ESP.^[^ ^38^ ^]^

To further investigate the neat film properties, we estimated the surface energies of PM6, Y6, and BPY using contact angle measurements using deionized water and diiodomethane (Figure S17 and Table S2, Supporting Information). Consequently, BPY exhibited a higher surface energy of 46.6 mJ m^−2^ than Y6 (41.0 mJ m^−2^), resulting in relatively low miscibility with PM6, which is consistent with the ESP calculation results.

**Figure 5:**
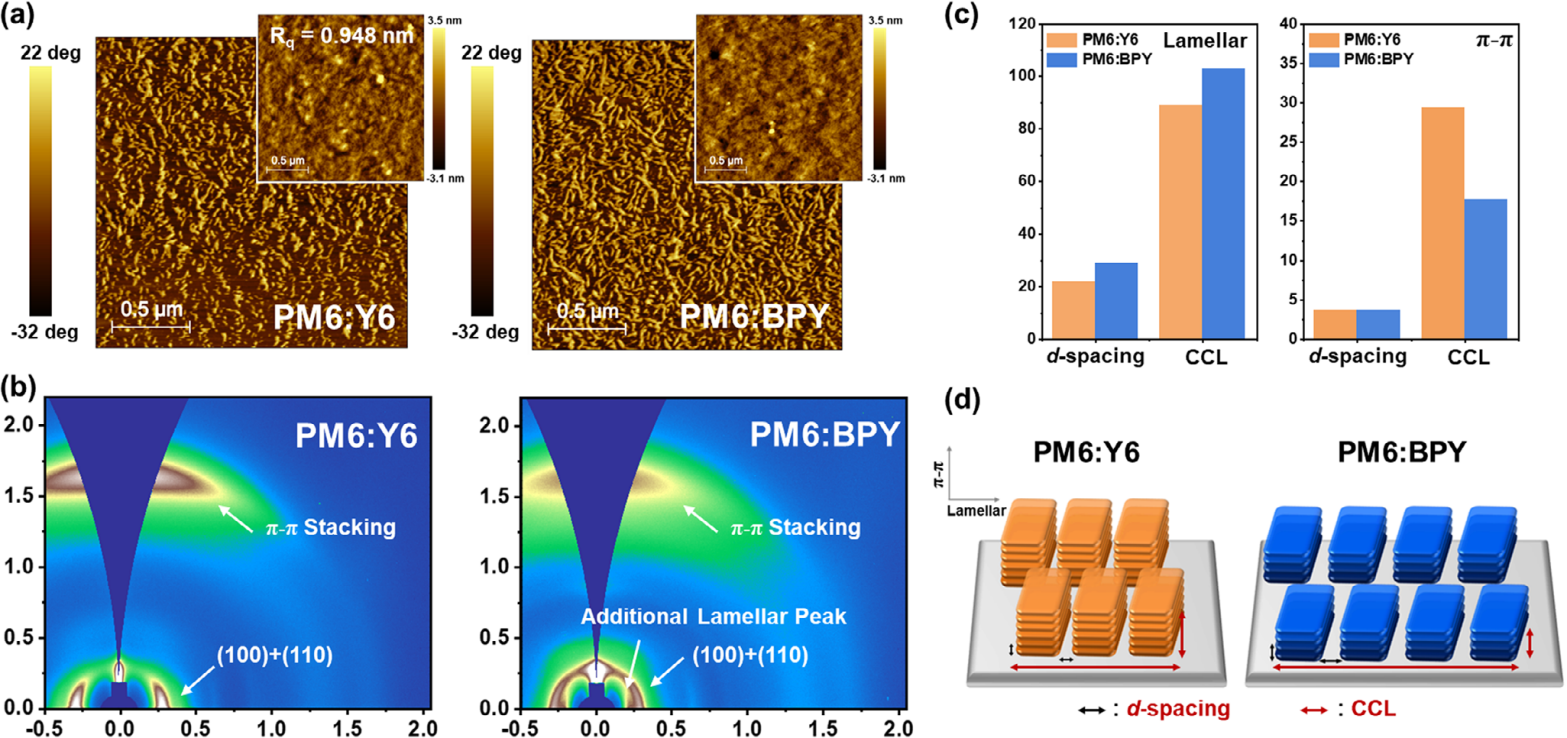
a) AFM phase and height (upper right) images of PM6:Y6 and PM6:BPY blend films. b) 2D GIXD imagesI of PM6:Y6 and PM6:BPY blend films. c) Histogram of d‐spacing and CCL of PM6:Y6 and PM6:BPY blend films for lamellar and π–π stackings. d) Schematic illustration of packing characteristics of PM6:Y6 and PM6:BPY blend films.

The morphology of blend films significantly influences OSC performance. AFM was used to analyze surface morphology. As shown in **Figure** **5**a, the PM6:Y6 and PM6:BPY films exhibited uniform and well‐intermixed surfaces with nearly identical surface roughness. This result indicates that the strong aggregation observed in the neat BPY film does not adversely affect the morphology of the blend film. This can be attributed to the higher dipole moment of BPY.^[^ ^31^ ^]^ In addition, the PM6:BPY film exhibits a clearer and more uniform fibrillar network in the phase images, which enhances exciton diffusion and reduces nonradiative recombination.^[^ ^39^, ^40^, ^41^ ^]^ GIXD was used to characterize the molecular packing behavior of the blend films. As shown in Figure 5b, both blend films exhibit a pronounced face‐on orientation. In the IP direction, the (100) peak for PM6:BPY appeared at a lower *q* value of 0.22 Å^−1^, corresponding to a *d*‐spacing of 29.15 Å. The *d*‐spacing of PM6:BPY is significantly larger than 21.78 Å of PM6:Y6. In the OOP direction, the (010) peaks for PM6:Y6 and PM6:BPY exhibited similar *d*‐spacings of approximately 3.63 Å (Figure S18 and Table S3, Supporting Information). However, the π–π stacking CCL for PM6:BPY was 17.75 Å, markedly smaller than 29.39 Å for PM6:Y6. This result suggests that the molecular packing in PM6:BPY is more loosely arranged, which facilitates the reduction of nonradiative energy loss in OSCs (Figure 5c,d).^[^ ^39^, ^42^ ^]^

### 2.6 Film Formation Kinetics

**Figure 6:**
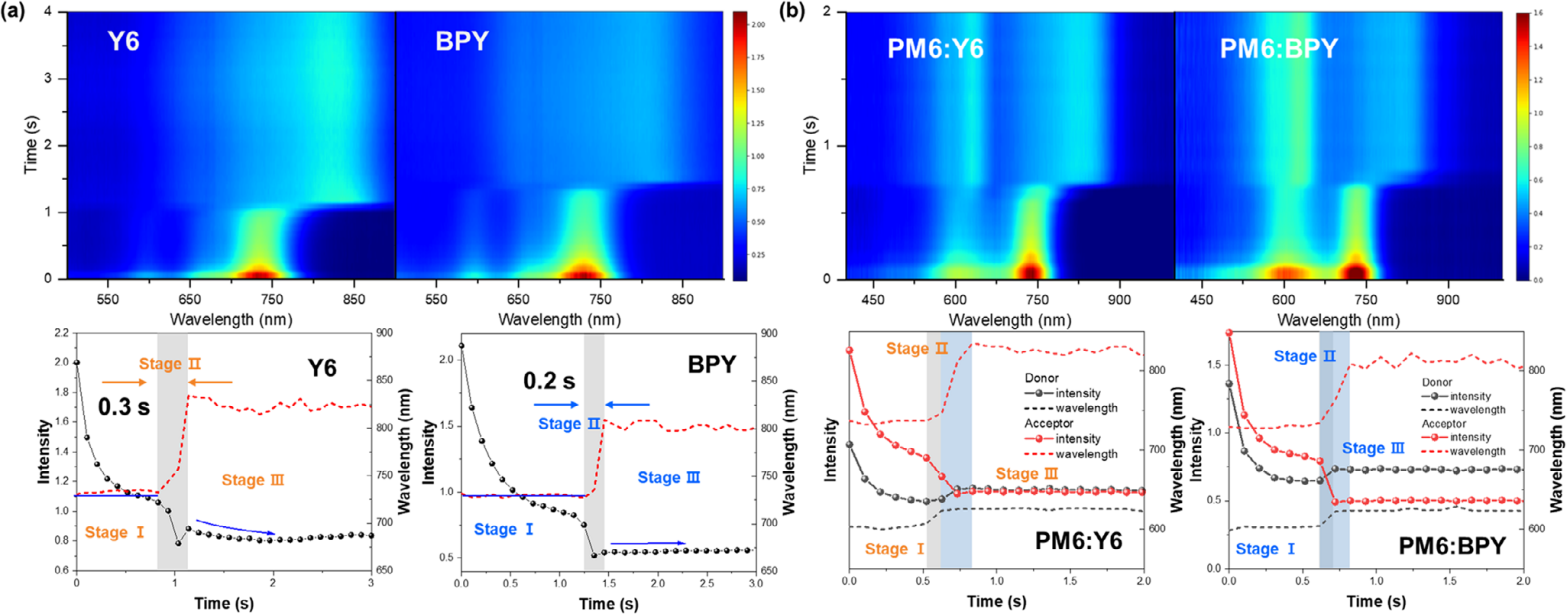
The 2D contour plots (top) and extracted values of absorption peak intensity and A_max_ chromic shift (bottom) in UV–vis spectra of a) Y6 and BPY and b) PM6:Y6 and PM6:BPY films.

We employed in situ UV–vis spectroscopy to monitor the formation of Y6 and BPY films in CF solutions (**Figures** **6**a and S19, Supporting Information). As the solvent rapidly evaporates, both materials exhibit three distinct stages of maximum absorption peak intensity and position: solution stage (Stage I), liquid–solid transition (Stage II), and film stage (Stage III). In Stage I, liquid film thinning under solvent evaporation and centrifugal force results in a pronounced decrease in peak intensity. During this period, Y6 exhibits a slight red shift, suggesting that solvent loss promotes initial molecular aggregation, whereas the absorption peak of BPY remains almost unchanged, indicating that increased solvent concentration have little effect on BPY aggregation. In Stage II, accelerated molecular aggregation and nucleation drive film solidification, accompanied by a pronounced red shift in the absorption peak. BPY completes this transition in approximately 0.2 s with a smaller wavelength shift, whereas Y6 undergoes a more significant red shift over approximately 0.3 s. By Stage III, the absorption intensity and wavelength stabilize, indicating the formation of solid films. Nevertheless, Y6 continues to exhibit gradual changes in peak intensity in the early part of Stage III, whereas BPY remains relatively steady. Overall, these results suggest that the presence of 3D side chains allows acceptors to achieve a stable morphology at an earlier stage.^[^ ^43^ ^]^

Figure 6b and Figure S20 (Supporting Information) also illustrate the film formation process for PM6 blended with either Y6 or BPY, which can be similarly divided into the same three stages based on the evolution of the donor and acceptor absorption peaks. In the PM6:Y6 blend, crystallization is first driven by donor aggregation for approximately 0.1 s, followed by acceptor aggregation over the next 0.2 s. This early donor aggregation provides Y6 with an additional nucleation time. In contrast, in the PM6:BPY blend, the donor and acceptor crystallize almost simultaneously: the donor completes aggregation in approximately 0.1 s, whereas the acceptor aggregation extends for approximately 0.2 s. Compared with PM6:Y6, this synchronized crystallization in PM6:BPY effectively reduces the risk of large‐scale phase separation. Although BPY and Y6 require approximately 0.2 s to aggregate, the absorption peak of Y6 shifts more dramatically, indicating a faster crystallization rate. Overall, the rapid solidification of BPY during film formation helps prevent excessive aggregation and phase separation in the blend film, thereby promoting favorable nanoscale morphology of the active layer and reducing nonradiative recombination losses in OSCs.^[^ ^44^ ^]^

## 3 Conclusion

In summary, we fabricated a Y‐series NFA named BPY by incorporating 3D conjugated nonflat biphenyl side chains into the outer position of the core framework. In‐depth investigations, including optical and theoretical analyses, demonstrate that the nonflat biphenyl conformation in BPY induces strong intermolecular interactions due to enhanced dipole moments and tuned ESP distribution, accompanied by partially isolated negative charges on the second phenyl rings. When BPY is blended with PM6, in addition to efficient charge dynamics via optimal fibrillar nanoscale surface morphology, the weakened π–π stacking minimizes energy loss. Consequently, these desirable features contribute to the higher PCE of the BPY‐based device than that of the Y6‐based device. In addition, by integrating BPY into a PM6:BTP‐eC9‐based OSC, a notable PCE of 19.23% was obtained, which was driven by simultaneous enhancements in *V*_OC_ and FF. This work demonstrates that the incorporation of biphenyl side chains can be a facile molecular design strategy for refining nonradiative energy loss in OSCs and realizing high‐performance optoelectronic devices.

## Conflict of Interest

The authors declare no conflict of interest.

## Supporting information



Supporting Information

## Data Availability

The data that support the findings of this study are available from the corresponding author upon reasonable request.
