# Supplementary material for: 3D Conjugated Nonflat Biphenyl Side Chains: Their Exclusive Role in Inducing Negative Electrostatic Potential in Efficient Organic Solar Cells
Source: Small. 2025 Aug 28;21(41):e09667. doi: 10.1002/smll.202509667 (PMC12530028; doi:10.1002/smll.202509667)
Supplement: Supplementary file 1 — Supporting Information [file SMLL-21-e09667-s001.docx]

Supporting Information

3D Conjugated Nonflat Biphenyl Side Chains: Their Exclusive Role in Inducing Negative Electrostatic Potential in Efficient Organic Solar Cells

Seonghun Jeong^†[a]^, Zhe Sun^†[a]^, Yongjoon Cho^[a]^, Sangjin Yang^[a]^, Thi Le Huyen Mai^[a]^, and Changduk Yang*^[a]^, ^[b]^

[a] Dr. S. Jeong, Z. Sun, Dr. Y. Cho, S. Yang, T. L. H. Mai, Prof. C. Yang
School of Energy and Chemical Engineering, Ulsan National Institute of Science and Technology (UNIST), 50 UNIST-gil, Ulju-gun, Ulsan 44919, South Korea.
E-mail : yang@unist.ac.kr

[b] Prof. C. Yang
Graduate School of Carbon Neutrality, Ulsan National Institute of Science and Technology (UNIST), 50 UNIST-gil, Ulju-gun, Ulsan 44919, South Korea.

*^[†]^* These authors contributed equally to this work.

**Experimental Section**

**Materials and instruments**

3-bromothieno[3,2-*b*]thiophene and 4,7-dibromo-5,6-dinitrobenzo[c][1,2,5]thiadiazole were synthesized according to previously reported methods. The other chemicals and reagents were purchased from Sigma-Aldrich, Tokyo Chemical Industry Co., Ltd, and Alfa Aesar Chemical Company and used without further purification, and 2-(5,6-difluoro-3-oxo-2,3-dihydro-1*H*-inden-1-ylidene)malononitrile was purchased from SunaTech Inc. All solvents are ACS and anhydrous grade by distillation. ^1^H NMR and ^13^C NMR spectra of the materials were recorded on a Bruker AVANCE III HD 400 MHz spectrometer using deuterated CDCl_3_ as solvent and tetramethylsilane (TMS) as an internal standard. For UV-Vis-NIR spectra of the Y6 and BPY, their solutions (4.00 mg mL^-1^ in CF and p-xylene) were used, and the films were prepared by deposition from the 4.00 mg mL^-1^ concentrated CF solution *via* the spin-casting method at 700 rpm. The optical bandgaps were estimated from the absorption onset of the as-cast thin films. CV measurements were performed on an Iviumstat.h with a three-electrode cell system in a nitrogen-bubbled 0.1 M tetra-*n*-butylammonium hexafluorophosphate (*n*-Bu_4_NPF_6_) solution in acetonitrile at a scan rate of 100 mV^−1^ s^−1^ at room temperature. An Ag/Ag^+^ electrode, platinum wire, and material-coated glassy carbon electrode were used as the reference electrode, counter electrode, and working electrode, respectively. The Ag/Ag^+^ reference electrode was calibrated using an Fc/Fc^+^ redox couple as an external standard, whose oxidation potential was set at −4.8 eV with respect to the zero-vacuum level. HOMO and LUMO energy levels of PM6, Y6, and BPY were obtained from the equation HOMO (eV) = − (*E*_ox_^onset^ – *E*_Fc_^onset^ + 4.8) and LUMO (eV) = − (*E*_red_^onset^ − *E*_Fc_^onset^ + 4.8). The decomposition and melting temperatures were determined by a thermogravimetric analyzer Q500 (TA Instruments) and a differential scanning calorimeter Q200 (TA Instruments), respectively.

The contact angles of PM6, Y6, and BPY were obtained using the Phoenix 300 Model instrument. AFM images of thin films were obtained using a multimode V microscope (Veeco, USA) with a nanoscope controller using Si tips (Bruker), and TEM analysis was performed using a JEOL USA JEM-2100F (Cs corrector) transmission electron microscope. Grazing incidence wide-angle X-ray scattering (GIXD) measurement was conducted at the PLS-II 9A and UNIST 6D beamline of the Pohang Accelerator Laboratory in Korea. The X-rays coming from the in-vacuum undulator were monochromated (λ = 1.10994 Å) using a double crystal monochromator and focused both horizontally and vertically (450 (H) × 60 (V) *µ*m^2^ in FWHM (full width at half maximum) @ the sample position) using K–B type mirrors. The GIXD sample stage was equipped with a 7-axis motorized stage for the fine alignment of the sample, and the incidence angle of the X-ray beam was set to be 0.12° for the neat and blend films. The GIXD patterns were recorded with a 2D CCD detector (Rayonix SX165), and the X-ray irradiation time was 5–30 s, dependent on the saturation level of the detector. Diffraction angles were calibrated using a sucrose standard (monoclinic, P21, *a* = 10.8631 Å, *b* = 8.7044 Å, *c* = 7.7624 Å, and β = 102.938°) and the sample-to-detector distance was ≈231 mm. CCL was calculated according to the following Scherrer equation: CCL = 2π*K*/*Δ*_q_

In this equation, CCL is the crystal coherence length, *K* is a shape factor (0.9), and *Δ*_q_ is the FWHM of a diffraction peak.

**Material synthesis and characterization**

*Synthesis of* ***(4-hexylphenyl)trimethylstannane (1)***

To a two-necked round-bottomed flask, 1-bromo-4-hexylbenzene (5 g, 15.4 mmol) was dissolved in THF. At -78 ^o^C, n-BuLi (2.5M, 8 mL) was added dropwise. The reaction mixture stirred at -78^o^C for an hour, followed by the addition of trimethyltinchloride (4.3g, 21.56 mmol), and stirred overnight under argon protection. The reaction mixture was quenched with water and washed with brine. Then, the solution was dried over MgSO_4_ and concentrated under reduced pressure. The residue was used for the next synthesis without further purification.

*Synthesis of* ***4-bromo-4'-hexyl-1,1'-biphenyl (2)***

To a two-necked round-bottomed flask, **Compound 1** (3 g, 9.23 mmol), 1,4-dibromobenzene (840.7 mg, 3.40 mmol), and tris(o-tolyl)phosphine (337 mg, 1.11 mmol) were dissolved in anhydrous toluene (120 mL) and purged with argon for 15 min. Then, 254 mg of tris(dibenzylideneacetone)dipalladium(0) (0.28 mmol) were added to reaction mixture, then purged again with argon for 20 min. After that, the reaction mixture was stirred at 120 °C overnight. Water was added to quench the reaction, then the mixture was extracted with dichloromethane. Combined organic layer was dried with anhydrous MgSO_4_ and solvent was removed under reduced pressure. The residue was purified with column chromatography with hexane as an eluent to afford the colorless liquid (71%, 2.75 g). ^1^H NMR (400 MHz, CDCl_3_) 7.57–7.51 (m, 2H), 7.51–7.40 (m, 4H), 7.24 (d, *J* = 1.6 Hz, 2H), 2.64 (m, 2H), 1.64 (m, 2H), 1.42–1.23 (m, 6H), 0.88 (m, 3H).

*Synthesis of* ***(4'-hexyl-[1,1'-biphenyl]-4-yl)trimethylstannane (3)***

To a solution of **Compound 2** (1 g, 3.15 mmol) in anhydrous THF was added *n*-butyllithium in 2.5 M solution (1.64 mL, 4.10 mmol) at -78 °C under Ar atmosphere. The mixture was stirred at the same temperature for an hour, followed by the addition of trimethyltinchloride (879 mg, 4.41 mmol). The reaction mixture was quenched with water and washed with brine. Then, the solution was dried over MgSO_4_ and concentrated under reduced pressure. The residue was used for the next synthesis without further purification.

*Synthesis of* ***3-(4'-hexyl-[1,1'-biphenyl]-4-yl)thieno[3,2-b]thiophene (4).***

To a two-necked round-bottomed flask, **Compound 3** (1.4 g, 3.50 mmol), 3-bromothienothiophene (1.53 g, 6.98 mmol), and tris(o-tolyl)phosphine (127 mg, 0.42 mmol) were dissolved in anhydrous *o*-xylene (40 mL) and purged with argon for 15 min. Then, 96 mg of tris(dibenzylideneacetone)dipalladium(0) (0.10 mmol) were added to reaction mixture, then purged again with argon for 20 min. After that, the reaction mixture was stirred at 160 °C overnight. Water was added to quench the reaction, then the mixture was extracted with dichloromethane. Combined organic layer was dried with anhydrous MgSO_4_ and solvent was removed under reduced pressure. The residue was purified with column chromatography with hexane as an eluent to afford the white solid (71%, 2.75 g). ^1^H NMR (400 MHz, CDCl_3_) 7.84 (d, *J* = 8.3 Hz, 2H), 7.70 (m, 2H), 7.57 (m, 3H), 7.33 (d, J = 5.2 Hz, 1H), 7.28 (m, 3H), 2.66 (m, 2H), 1.65 (m, 2H), 1.43–1.25 (m, 6H), 0.90 (m, 3H).

*Synthesis of* ***(6-(4'-hexyl-[1,1'-biphenyl]-4-yl)thieno[3,2-b]thiophen-2-yl)trimethylstannane (5).***

To a solution of **Compound 4** (670 mg, 1.78 mmol) in anhydrous THF was slowly added Lithium diisopropylamide in 2 M solution (1.25 mL, 2.50 mmol) at -78 °C under Ar atmosphere. The mixture was stirred at the same temperature for an hour, followed by the addition of trimethyltinchloride (532 mg, 2.67 mmol). The reaction mixture was quenched with water and washed with brine. Then, the solution was dried over MgSO_4_ and concentrated under reduced pressure. The residue was used for the next synthesis without further purification.

*Synthesis of* ***4,7-bis(6-(4'-hexyl-[1,1'-biphenyl]-4-yl)thieno[3,2-b]thiophen-2-yl)-5,6-dinitrobenzo[c][1,2,5]thiadiazole (6).***

To a two-neck round-bottom flask of **compound 5** (930 mg, 1.72 mmol), 4,7-dibromo-5,6-dinitrobenzo[c][1,2,5]thiadiazole (288 mg, 0.75 mmol), and tetrakis(triphenylphosphine)palladium(0) (87 mg, 0.08 mmol) in 15 mL of toluene were added under argon protection. The reaction mixture was refluxed overnight with vigorous stirring. After cooling to room temperature, the solvent was removed under reduced pressure. The residue was purified by column chromatography to afford the purple solid (1.45 g, 91%). ^1^H NMR (400 MHz, CDCl_3_) 7.86–7.65 (m, 6H), 7.75 (m, 2H), 7.29 (m, 2H), 2.67 (m, 2H), 1.66 (m, 2H), 1.43–1.24 (m, 6H), 0.90 (m, 3H).

*Synthesis of* ***12,13-bis(2-butyloctyl)-3,9-bis(4'-hexyl-[1,1'-biphenyl]-4-yl)-12,13-dihydro-[1,2,5]thiadiazolo[3,4-e]thieno[2'',3'':4',5']thieno[2',3':4,5]pyrrolo[3,2-g]thieno[2',3':4,5]thieno[3,2-b]indole (7).***

**Compound 6** (700 mg, 0.72 mmol) and triethyl phosphite (10 mL) were dissolved in anhydrous *o*-dichlorobenzene (10 mL) under Ar atmosphere. The mixture was stirred and heated at 180 °C for 20 hours. After cooling to room temperature, the reaction mixture was extracted with brine and DCM and dried over magnesium sulfate. After concentrating the reaction mixture under reduced pressure, the residue was transferred to a two-neck round-bottom flask. Subsequently, the orange mixture was mixed with potassium carbonate (496 mg, 3.59 mmol), potassium iodide (596 mg, 3.59 mmol), and dimethylformamide (15 mL) under Ar atmosphere stirred at 80 °C for an hour. Then, 1-bromo-2-butyloctane (716 mg, 2.87 mmol) was added and stirred at 130 °C overnight. After cooling to room temperature, the reaction mixture was extracted with DI water and DCM, and dried over magnesium sulfate. The residue was purified by column chromatography obtain the orange solid (405.9 mg, 38%). ^1^H NMR (400 MHz, CDCl_3_) 7.96 (d, *J* = 8.3 Hz, 4H), 7.75 (d, *J* = 8.3 Hz, 4H), 7.61 (d, *J* = 8.1 Hz, 4H), 7.55 (s, 2H), 7.31 (d, *J* = 8.1 Hz, 4H), 4.67 (d, *J* = 7.8 Hz, 4H), 2.68 (m, 4H), 1.67 (m, 4H), 1.42–0.83 (m, 52H), 0.64 (m, 12H).

*Synthesis of* ***12,13-bis(2-butyloctyl)-3,9-bis(4'-hexyl-[1,1'-biphenyl]-4-yl)-12,13-dihydro-[1,2,5]thiadiazolo[3,4-e]thieno[2'',3'':4',5']thieno[2',3':4,5]pyrrolo[3,2-g]thieno[2',3':4,5]thieno[3,2-b]indole-2,10-dicarbaldehyde (8).***

Anhydrous dimethylformamide (0.5 mL), phosphorus oxychloride (0.5 mL), and 1,2-dichloroethane (2 mL) were mixed at 0 °C in a two-neck round-bottom flask, and the mixture was stirred for one and half hour. Then, the mixture was transferred dropwise to the solution of **compound 7** (140 mg, 0.11 mmol) in 1,2-dichloroethane (10 mL) and stirred at 130 °C overnight. The reaction mixture was quenched with sodium hydrogen carbonate over three hours, extracted with DCM, and dried over magnesium sulfate. The residue was purified by column to afford the yellow solid (406.8 mg, 95%). ^1^H NMR (400 MHz, CDCl_3_) 10.06 (s, 2H), 7.86 (m, 8H), 7.64 (d, J = 8.0 Hz, 4H), 7.36 (d, J = 8.0 Hz, 4H), 4.72 (m, 4H), 2.71 (m, 4H), 1.71 (m, 4H), 1.43-0.82 (m, 52H), 0.68 (m, 12H).

*Synthesis of* ***2,2'-((2Z,2'Z)-((12,13-bis(2-butyloctyl)-3,9-bis(4'-hexyl-[1,1'-biphenyl]-4-yl)-12,13-dihydro-[1,2,5]thiadiazolo[3,4-e]thieno[2'',3'':4',5']thieno[2',3':4,5]pyrrolo[3,2-g]thieno[2',3':4,5]thieno[3,2-b]indole-2,10-diyl)bis(methaneylylidene))bis(5,6-difluoro-3-oxo-2,3-dihydro-1H-indene-2,1-diylidene))dimalononitrile (BPY)*.**

**Compound 8** (130 mg, 0.10 mmol) and 2-(5,6-difluoro-3-oxo-2,3-dihydro-1*H*-inden-1-ylidene)malononitrile (103.3 mg, 0.45 mmol) were added pyridine (1 mL) in 10 mL of chloroform. The mixture was stirred at 70 °C overnight. After cooling to room temperature, the reaction mixture was quenched with water, extracted with brine and chloroform, and dried over magnesium sulfate. After concentrating the reaction mixture under reduced pressure, the residue was purified by column chromatography with hexane and DCM, yielding a dark blue solid (453.8 mg, 80%). ^1^H NMR (400 MHz, CDCl_3_) 8.88 (s, 2H), 8.52 (dd, J = 10.0, 6.5 Hz, 2H), 7.86 (d, J = 8.1 Hz, 4H), 7.72 (dd, J = 10.5, 7.7 Hz, 6H), 7.65 (d, J = 7.9 Hz, 4H), 7.34 (d, J = 7.9 Hz, 4H), 4.82 (m, 4H), 2.70 (t, J = 7.7 Hz, 4H), 2.17 (m, 2H), 1.68 (m, 4H), 1.40-0.87 (m, 50H), 0.74-0.61 (m, 12H). ^13^C NMR (100 MHz, CDCl_3_) 185.80, 158.75, 151.37, 147.51, 144.85, 143.46, 143.08, 138.41, 137.64, 137.41, 136.77, 134.92, 134.52, 134.11, 133.51, 131.59, 130.91, 130.64, 129.10, 128.31, 127.20, 121.38, 114.93, 114.79, 114.55, 113.61, 113.58, 112.58, 112.46, 69.76, 55.74, 39.33, 35.74, 31.80, 31.65, 31.52, 30.63, 30.52, 30.48, 30.36, 29.74, 29.50, 29.46, 29.08, 28.20, 27.96, 25.46, 25.20, 22.91, 22.85, 22.68, 22.53, 22.51, 14.17, 14.07, 14.04, 13.86, 13.81.

**Morphological Characterization.** Atomic force microscopy (AFM) was carried out using a Bruker Dimension Icon in standard tapping mode, equipped with a Bruker TESPA-V2 Si cantilever. Grazing incidence wide-angle X-ray scattering (GIWAXS) measurements were performed at the PLS-II 6D beamline of the Pohang Accelerator Laboratory in Korea. The X-ray beam, generated by an in-vacuum undulator (IVU), was monochromated to a wavelength of 1.07220 Å using a double crystal monochromator. The beam was focused to 450 µm (H) × 60 µm (V) full width at half maximum (FWHM) at the sample position by Kirkpatrick-Baez (K-B) mirrors. The sample stage was equipped with a 7-axis motorized platform for precise alignment. The incidence angle of the X-ray beam was set to 0.12° to optimize scattering from the films.

**Device Fabrication and Characterization.** Organic solar cells were fabricated using a conventional ITO/PEDOT:PSS/active layer/H75/Ag architecture. Patterned ITO-coated glass substrates were sequentially cleaned with detergent, deionized water, acetone, and isopropanol, followed by drying in an oven overnight. Prior to use, the ITO substrates were exposed to ultraviolet-ozone for 20 minutes. PEDOT:PSS was spin-coated at 4000 rpm for 60 s and annealed at 150 °C for 15 minutes. Substrates were transferred into a nitrogen-filled glove box for subsequent processing. A blend of PM6:NFA (1:1.2 weight ratio) was dissolved in chloroform at a total concentration of 15 mg mL^-1^ and stirred for 1 hour at room temperature. 1-chloronaphthalene (CN, 0.5 v/v%) was added as the processing additive. The solution was spin-coated onto PEDOT:PSS layers at 2400 rpm for 30 s, followed by thermal annealing at 100 °C for 5 minutes. A methanol solution of H75 (2.0 mg mL^-1^) was spin-coated at 3000 rpm for 30 s. A 100 nm Ag layer was thermally evaporated under high vacuum (<3.0 × 10^-4^ Pa) as the top electrode. The device active area was 4.3 mm². Current density-voltage (*J*–*V*) measurements were performed using a Keithley 2400 source meter under a simulated AM 1.5G illumination (SX1100, Enlitech) calibrated by a Newport 91150-KG5 reference cell. External quantum efficiency (EQE) was measured using an Enlitech QE-R3011 system, and electroluminescence quantum efficiency (EQE_EL_) was recorded by driving the devices with an external voltage source (ELCT-3010, Enlitech).

**Space-Charge-Limited Current (SCLC) Method.** Hole-only devices were fabricated with the structure ITO/PEDOT:PSS/active layer/MoO_3_, while electron-only devices used the configuration ITO/ZnO/active layer/H75/Ag. The SCLC mobilities were determined by fitting the *J*–*V* curves to the Mott-Gurney law:

$$J=\frac{9\varepsilon_{0}\varepsilon_{r}\mu V^{2}}{8L^{3}}$$

where ε_0_ is the vacuum permittivity, ε_r_ is the relative dielectric constant of the transport layer (assumed to be 3 for organic semiconductors), μ is the charge carrier mobility, V is the effective voltage, and L is the active layer thickness. Measurements were repeated to ensure reproducibility across multiple devices.

**Exciton dissociation efficiency (η_diss_) and charge collection efficiency (η_coll_).** The photocurrent density (*J*_ph_) was calculated as the difference between the current density under illumination (*J*_L_) and in the dark (*J*_D_), expressed as *J*_ph_ = *J*_L_ − *J*_D_. The effective voltage (*V*_eff_) was determined by the relation *V*_eff_ = *V*_0_ – *V*_app_, where *V*_0_ represents the built-in voltage, corresponding to the point where *J*_ph_ reaches zero, and *V*_app_ is the externally applied bias voltage. η_diss_ was derived from the ratio η_diss_ = *J*_ph_^sc^ / *J*_sat_, where *J*_ph_^sc^ is the photocurrent density at short circuit (V = 0) and *J*_sat_ is the saturated photocurrent density under high reverse bias. η_coll_ was evaluated using η_coll_ = *J*_ph_^max^ / *J*_sat_, where *J*_ph_^max^ corresponds to the photocurrent density at the maximum power point (*V*_max_) and *J*_sat_ remains the saturated photocurrent.


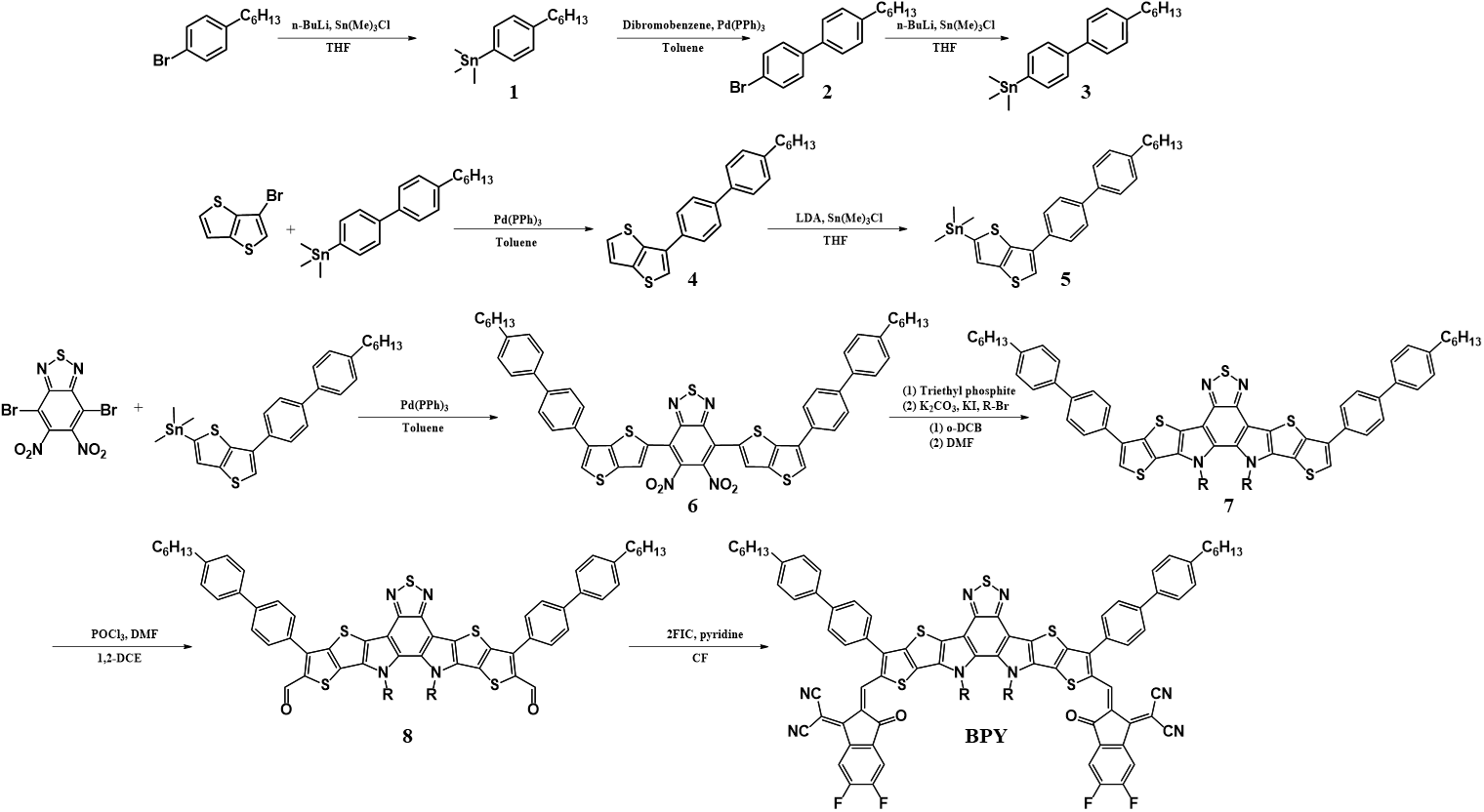


**Scheme 1.** Synthetic route for BPY.


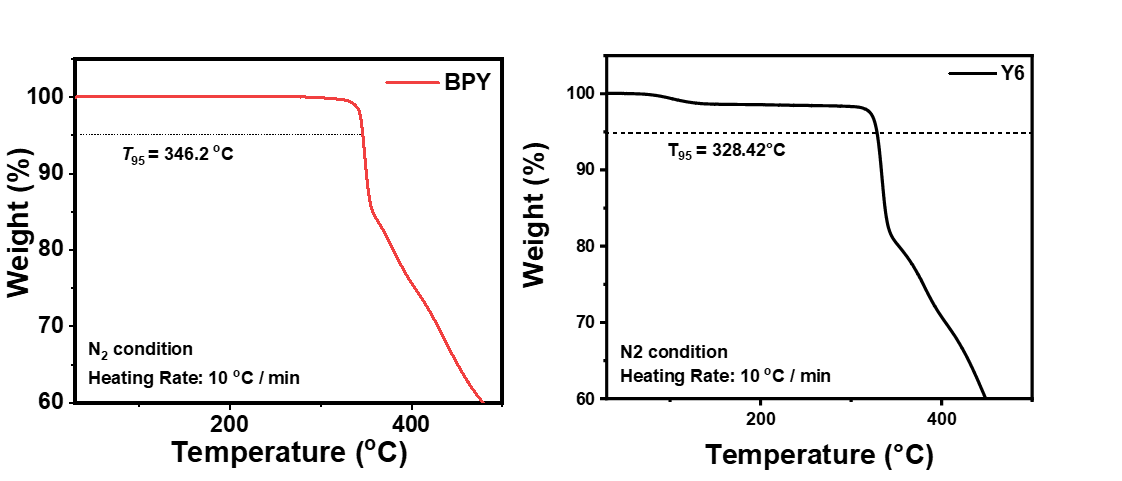


Figure S1. TGA plots of BPY and Y6.

Figure S2. DSC plots of BPY and Y6.

**
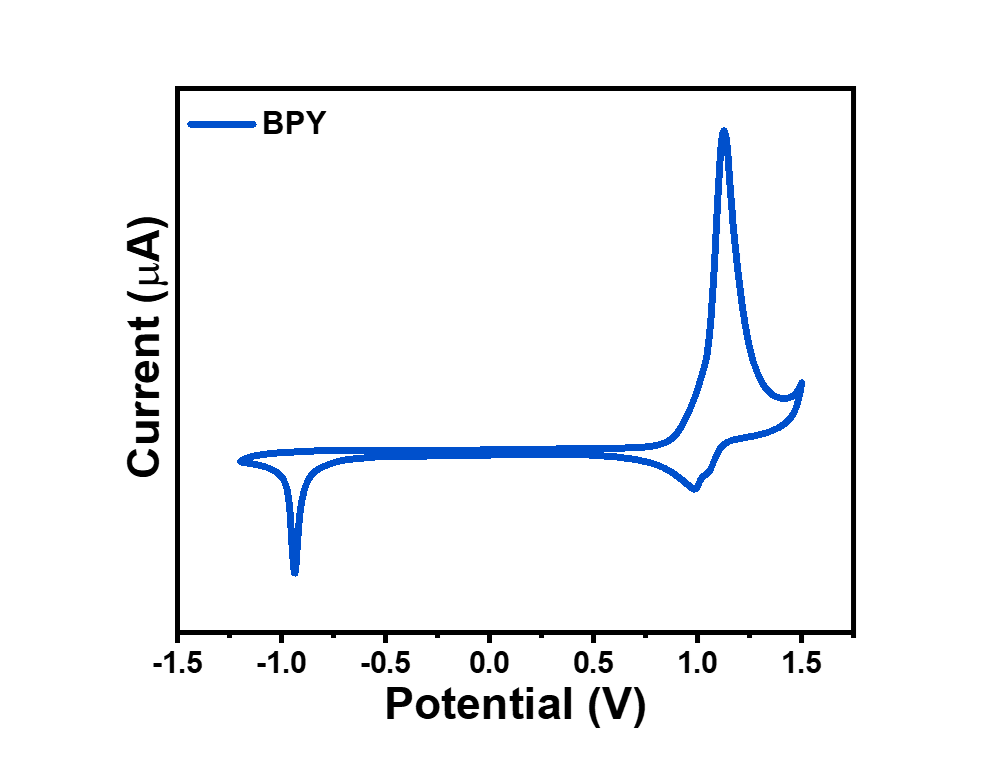
**

**Figure S3.** Result of cyclic voltammetry of BPY.


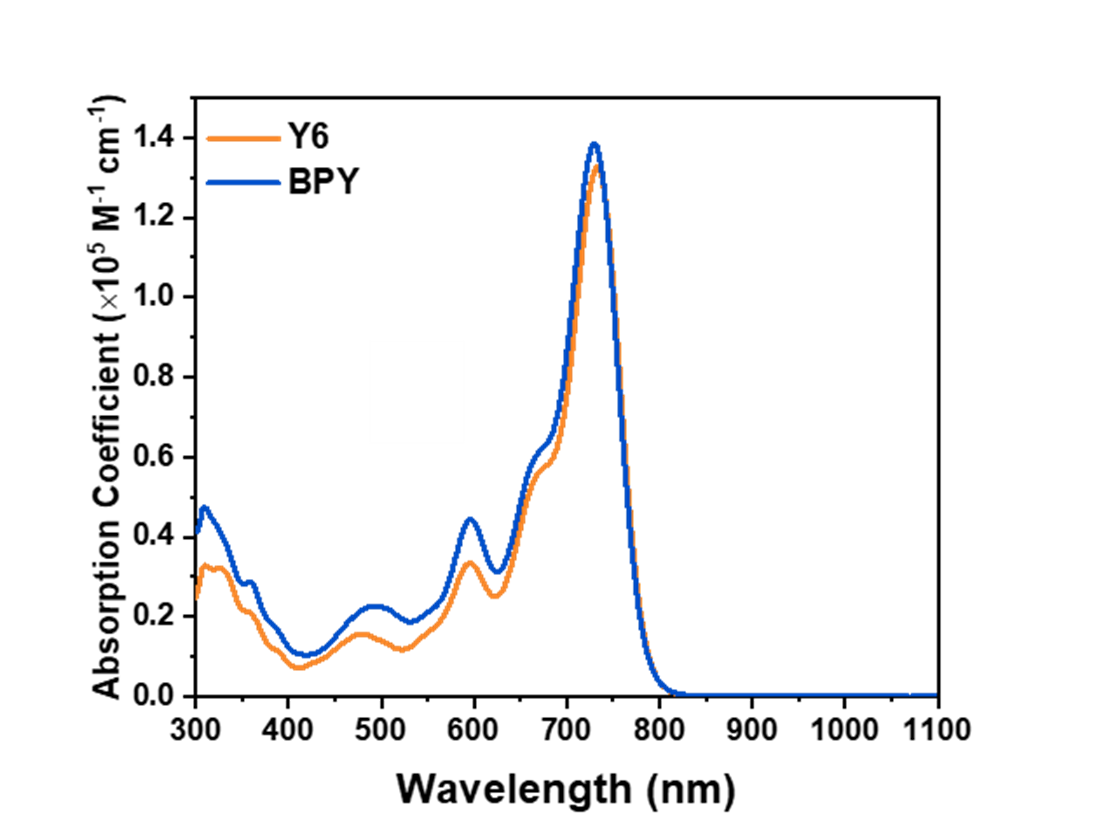


**Figure S4.** Absorption coefficient of Y6 and BPY in chloroform solutions.

**
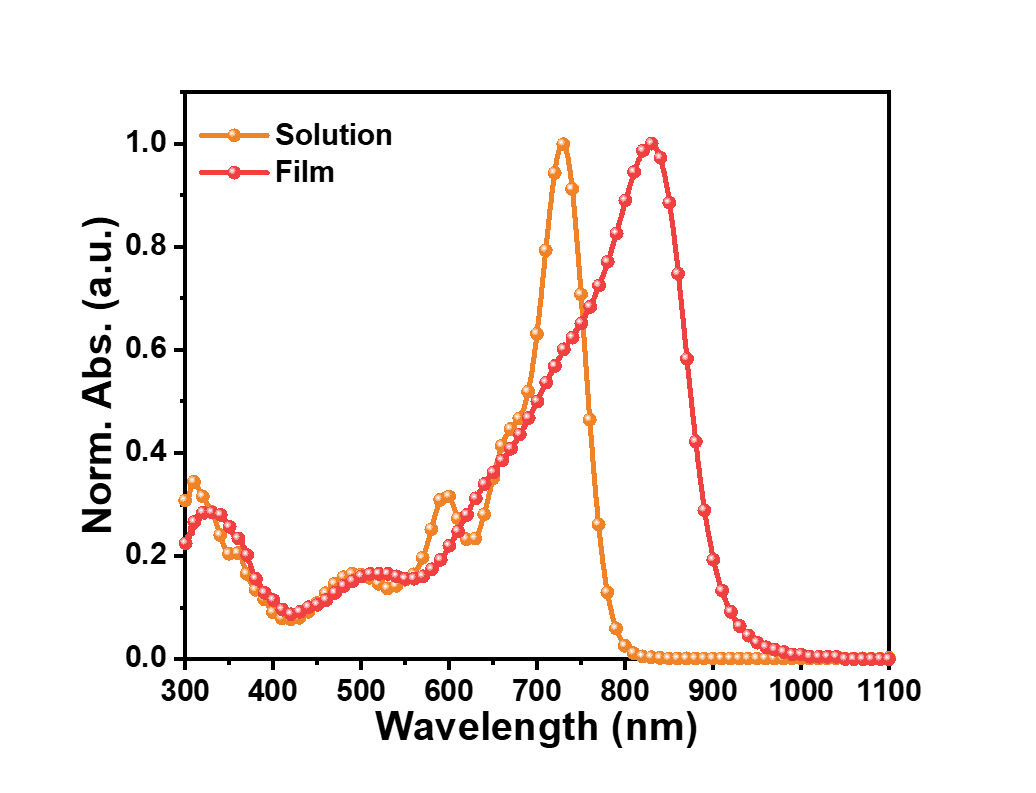
**

**Figure S5**. UV-vis absorption spectra of Y6 in chloroform solution and film state.


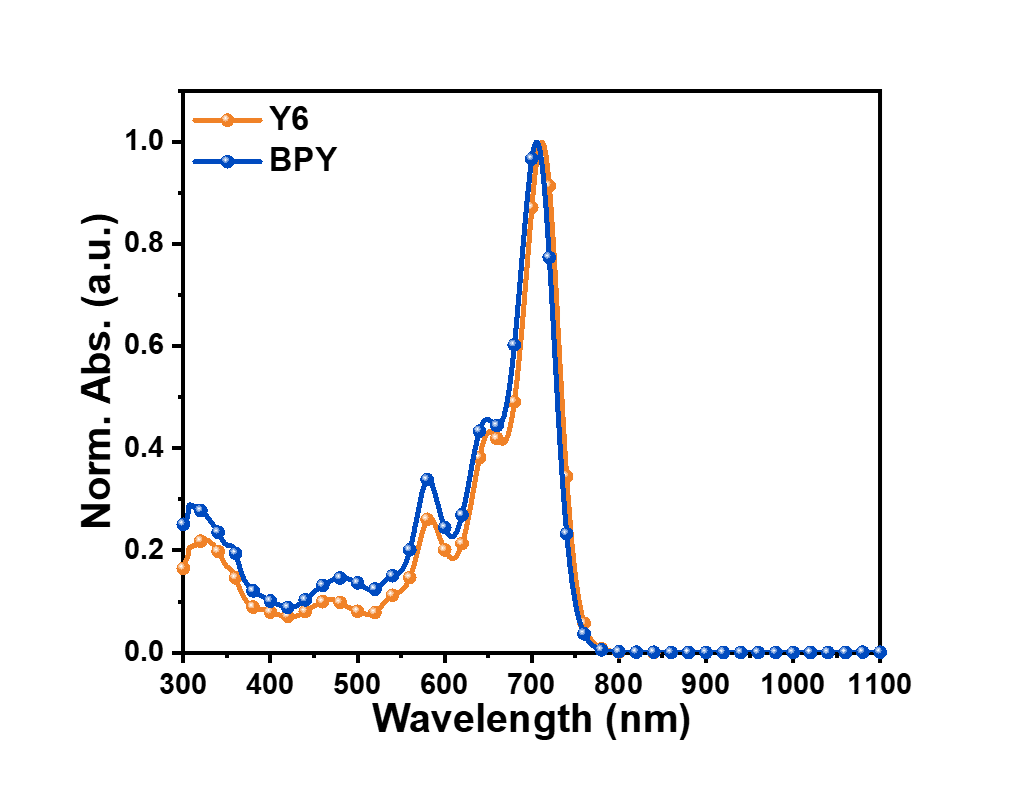


Figure S6. UV-vis absorption spectra of Y6 and BPY in *p*-xylene solution.


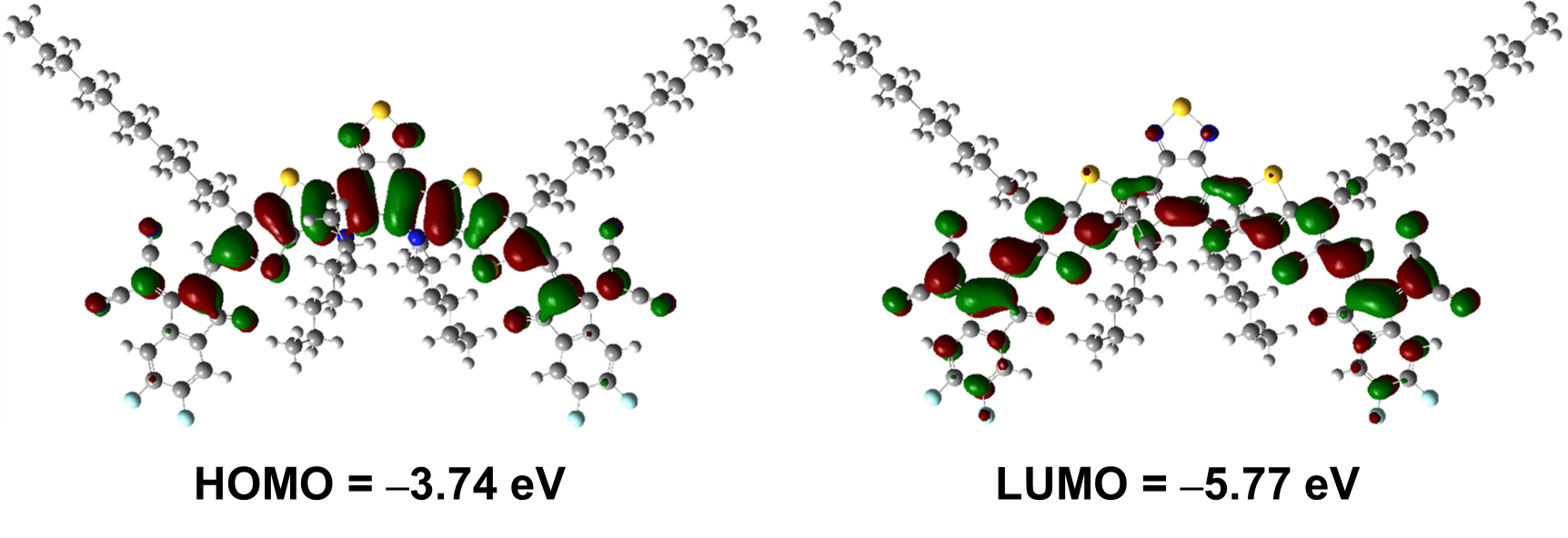


Figure S7. Frontier orbitals distribution of Y6 in optimized molecular geometry.


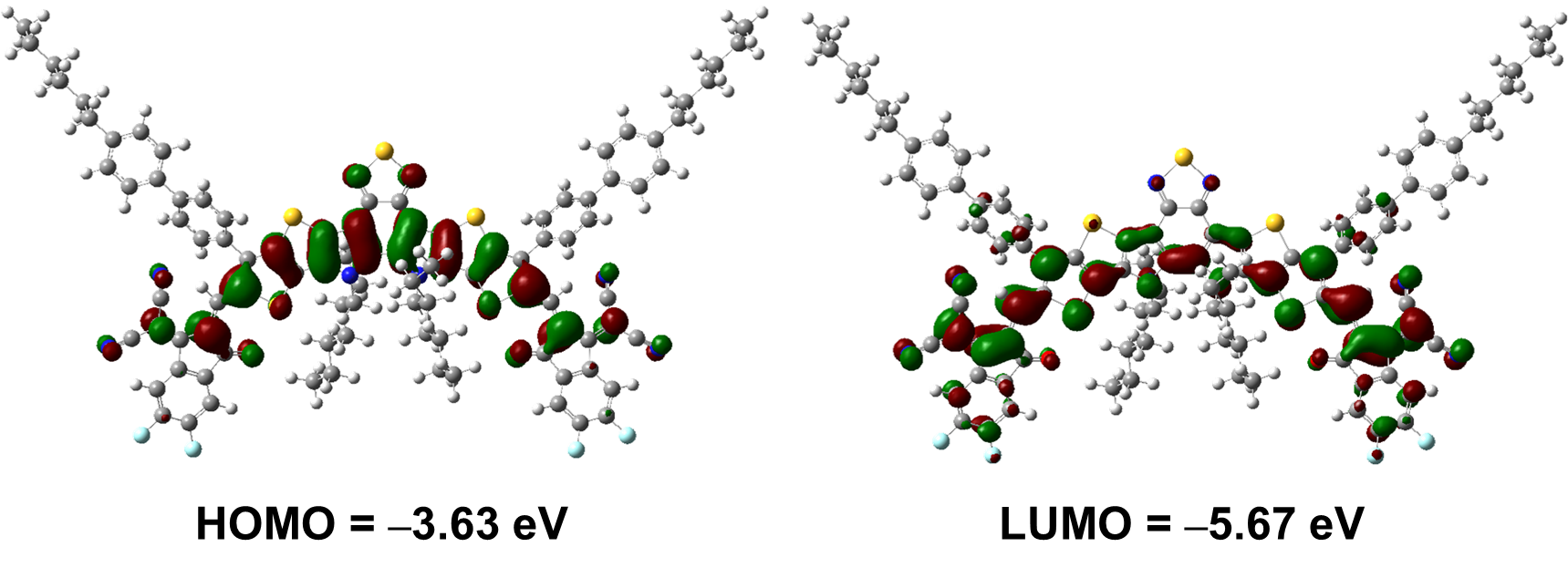


Figure S8. Frontier orbitals distribution of BPY in optimized molecular geometry.


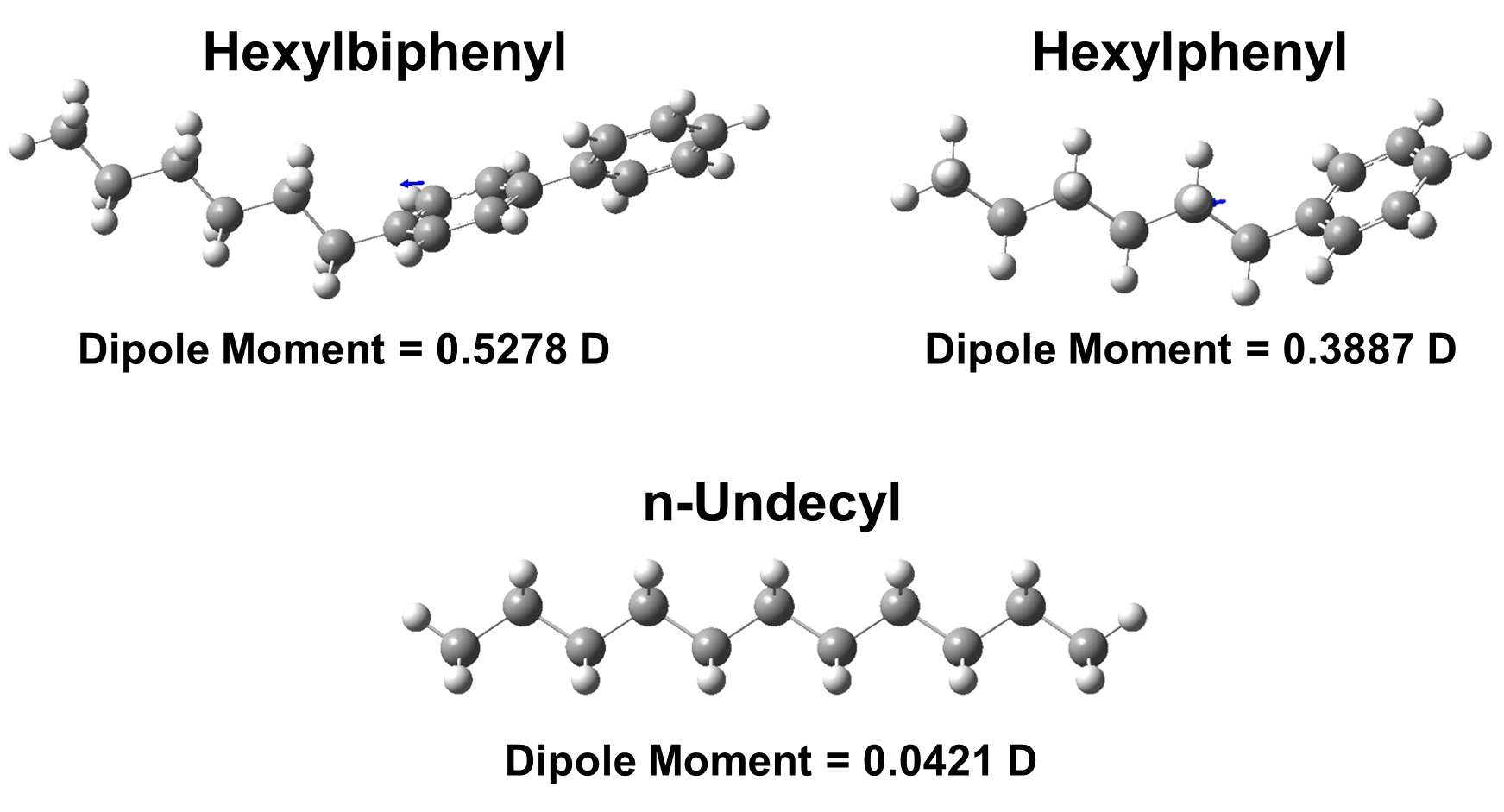


Figure S9. Local dipole moment of hexylbiphenyl, n-undecyl, and hexylphenyl side chains.


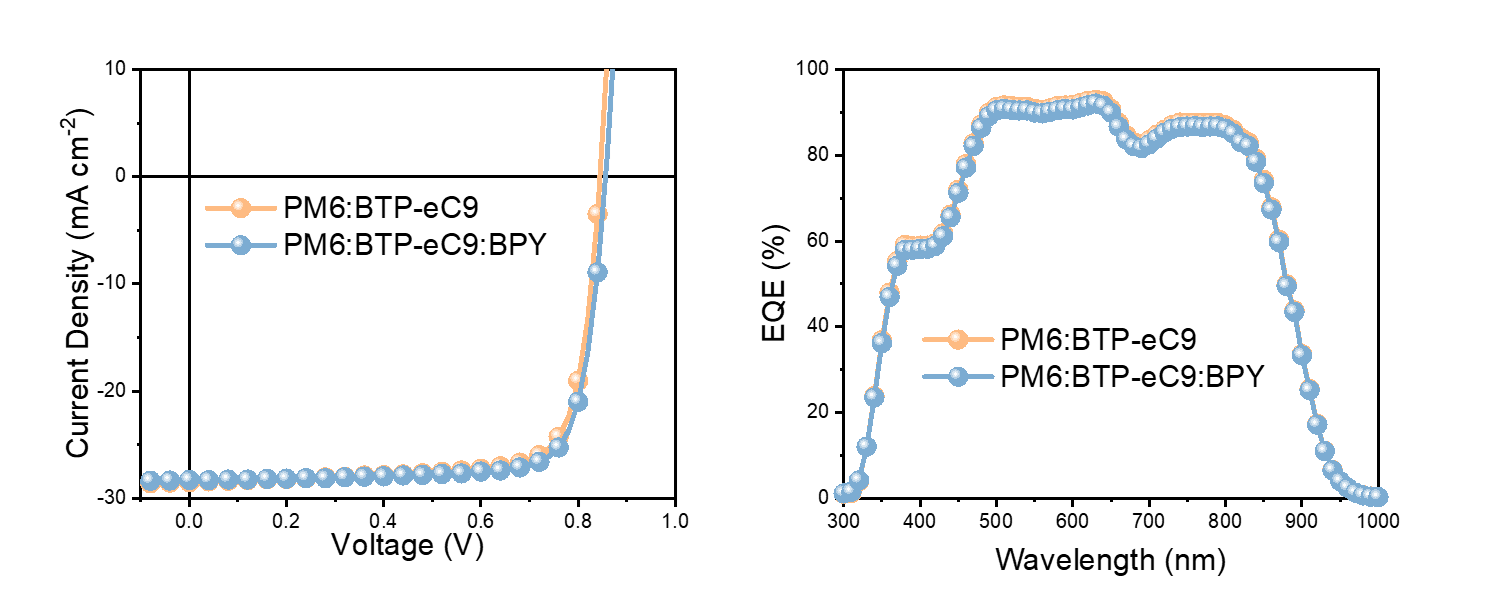


Figure S10. J-V curves and EQE spectra of PM6:BTP-eC9 and PM6:BTP-eC9:BPY-based OSCs.

Figure S11. Normalized PCE versus time for PM6:BPY and PM6:Y6 devices at 65 ℃ (average of 5 devices).


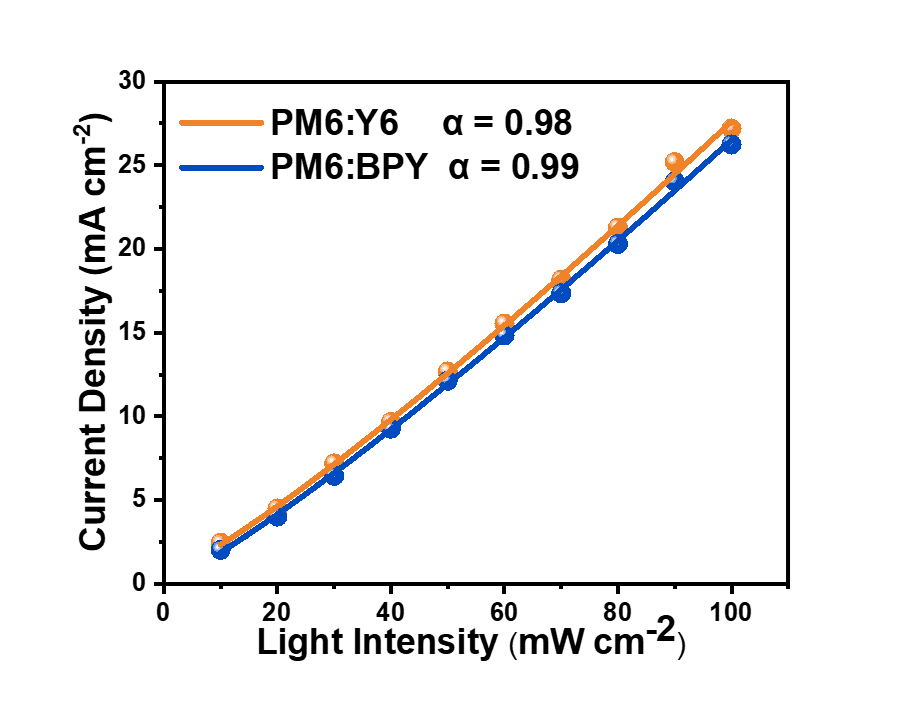


Figure S12. Light intensity dependence of J_SC_ for the optimized OSCs based on PM6:Y6 and PM6:BPY.


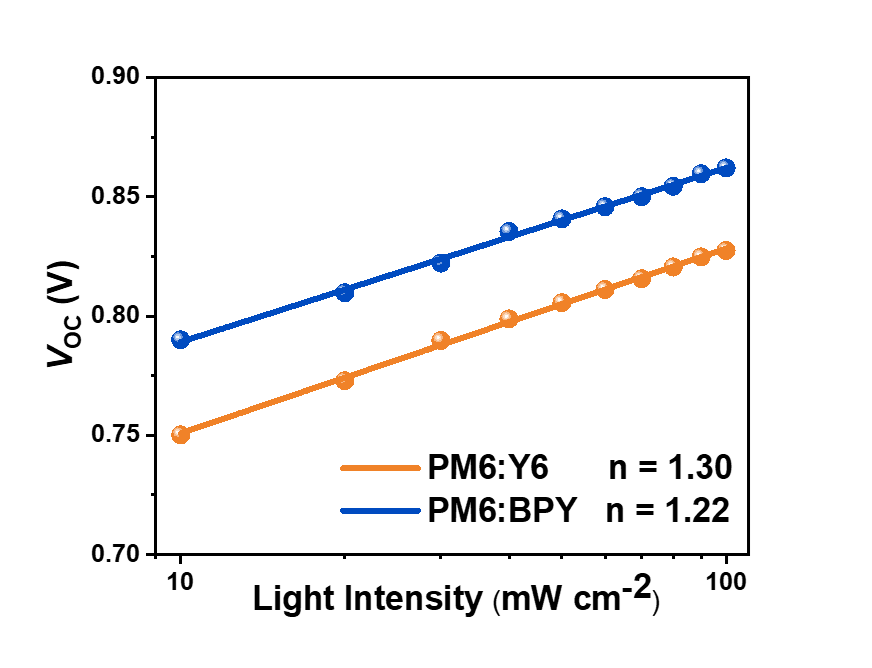


Figure S13. Light intensity dependence of V_OC_ for the optimized OSCs based on PM6:Y6 and PM6:BPY.


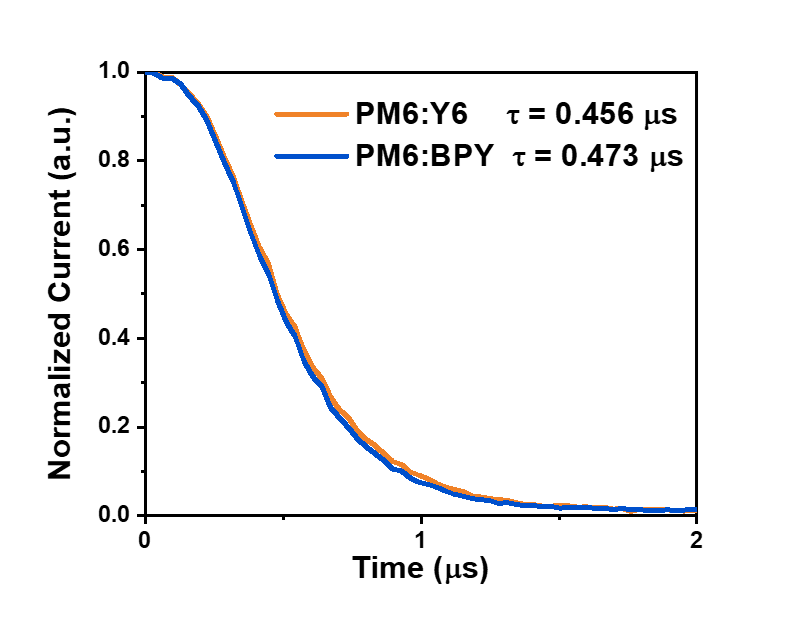


Figure S14. TPC plots for optimized OSCs based on PM6:Y6 and PM6:BPY.


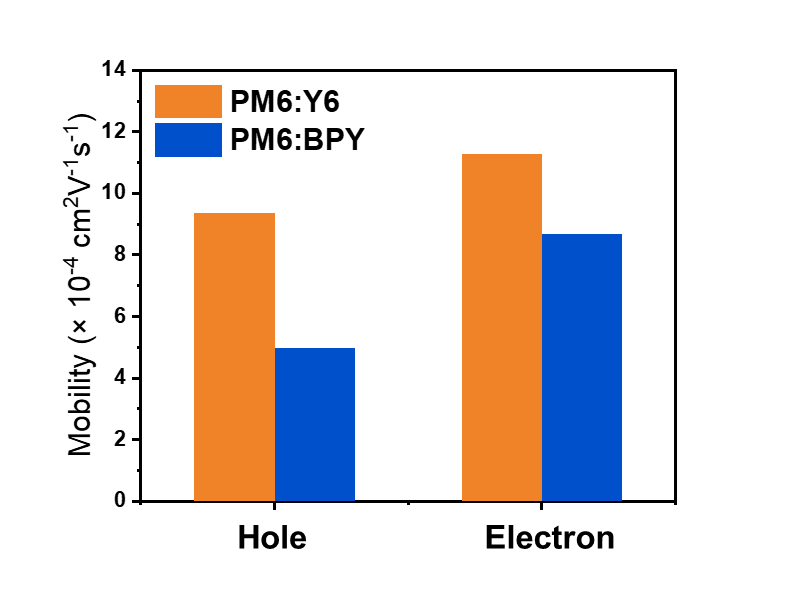


Figure S15. SCLC mobilities of optimized OSCs based on PM6:Y6 and PM6:BPY.


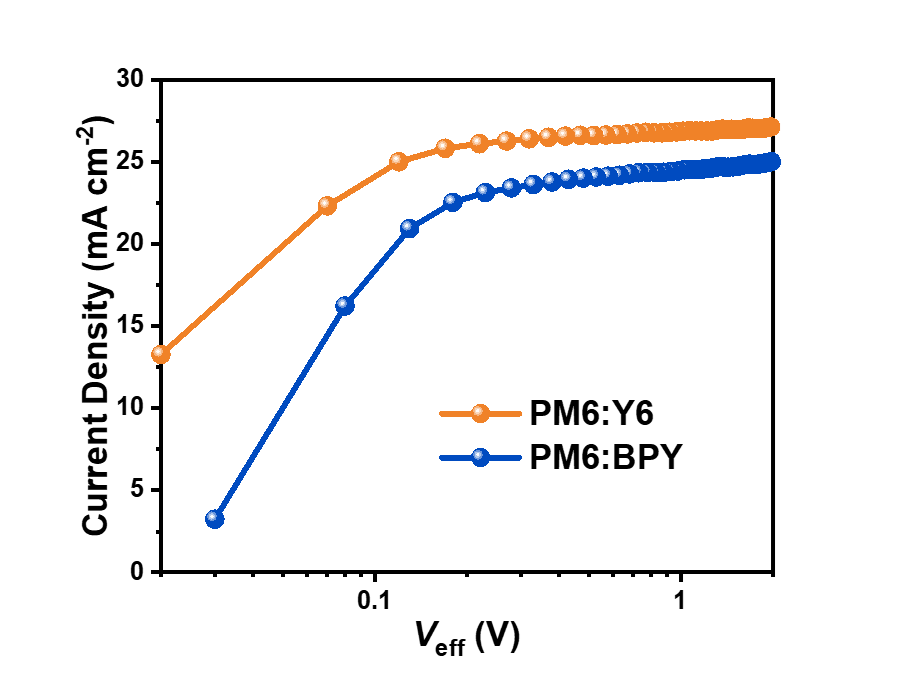


Figure S16. J_Ph_–V_eff_ curves of optimized OSCs based on PM6:Y6 and PM6:BPY.

**Table S1.** Crystallographic parameters of Y6 and BPY neat films.


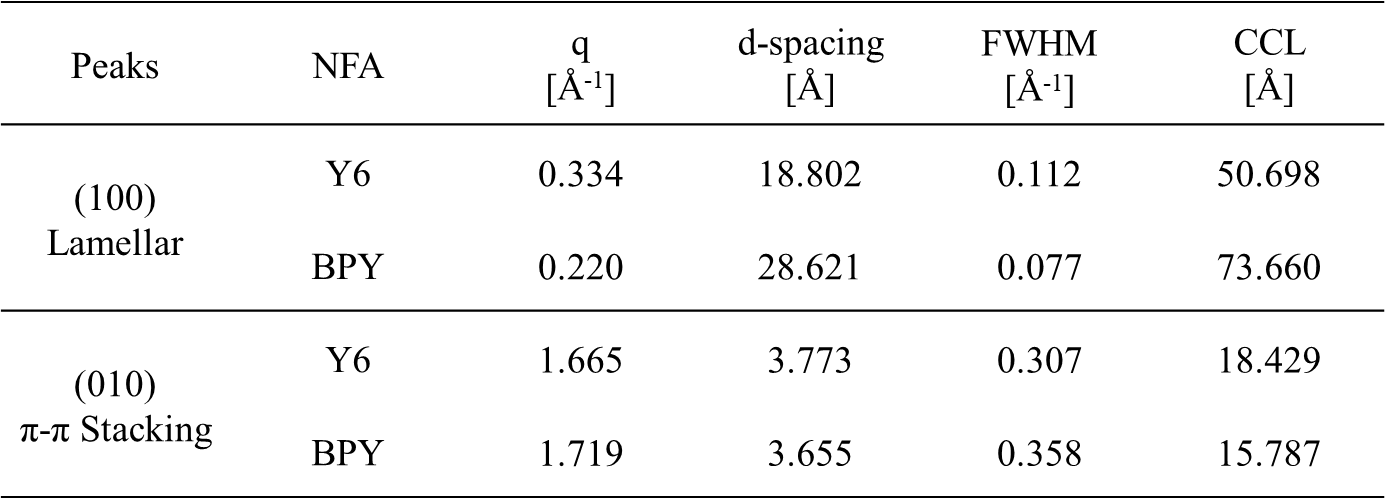


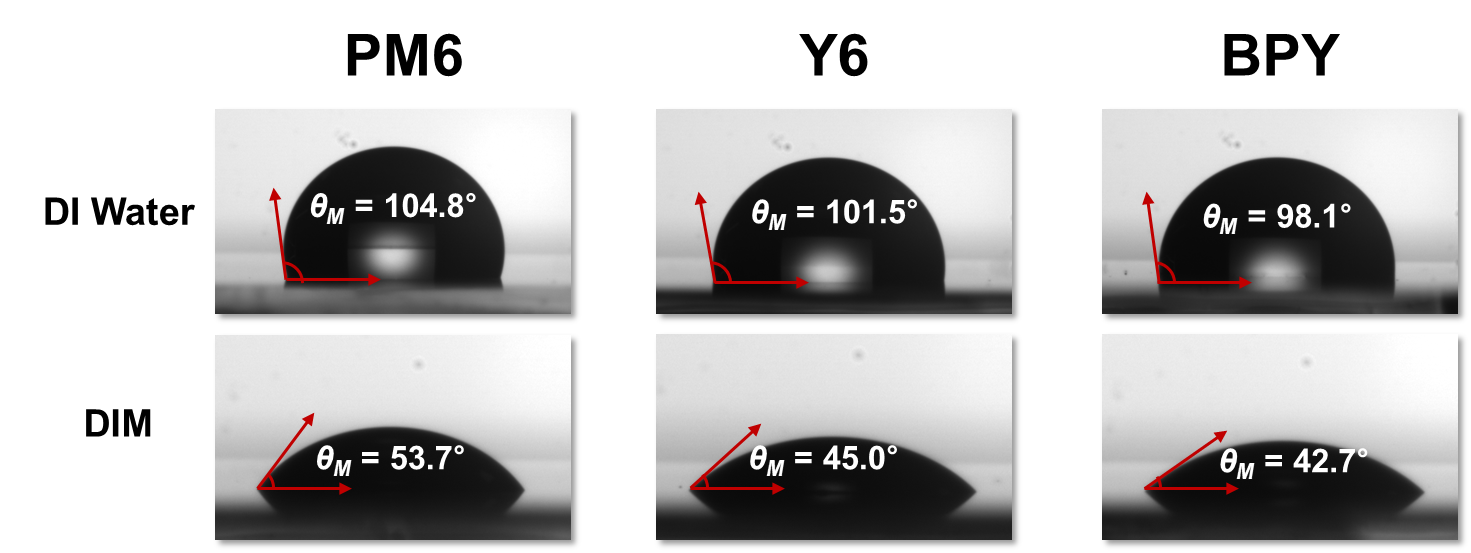


Figure S17. Contact angles of PM6, Y6, and BPY.

**Table S2.** Contact angles and surface energies of PM6, Y6, and BPY films.


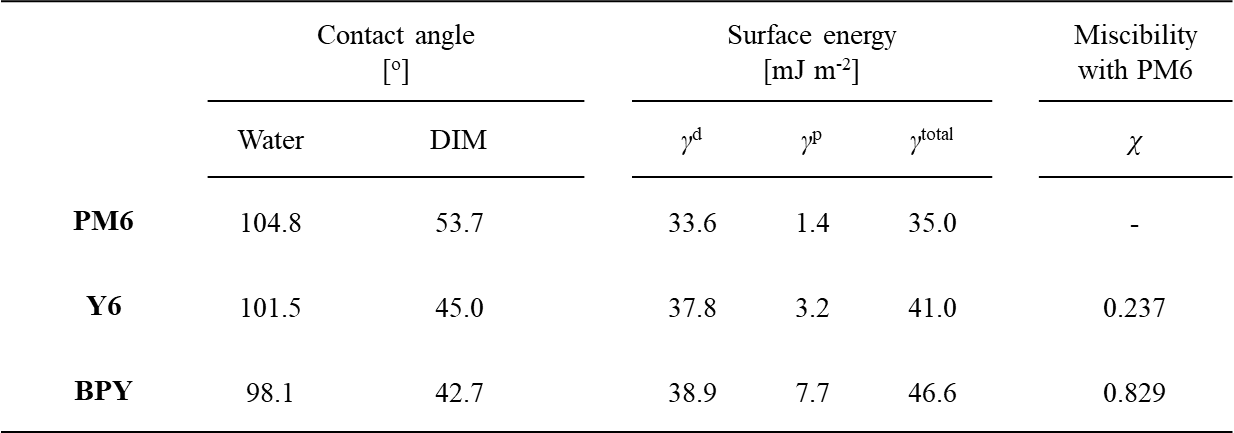


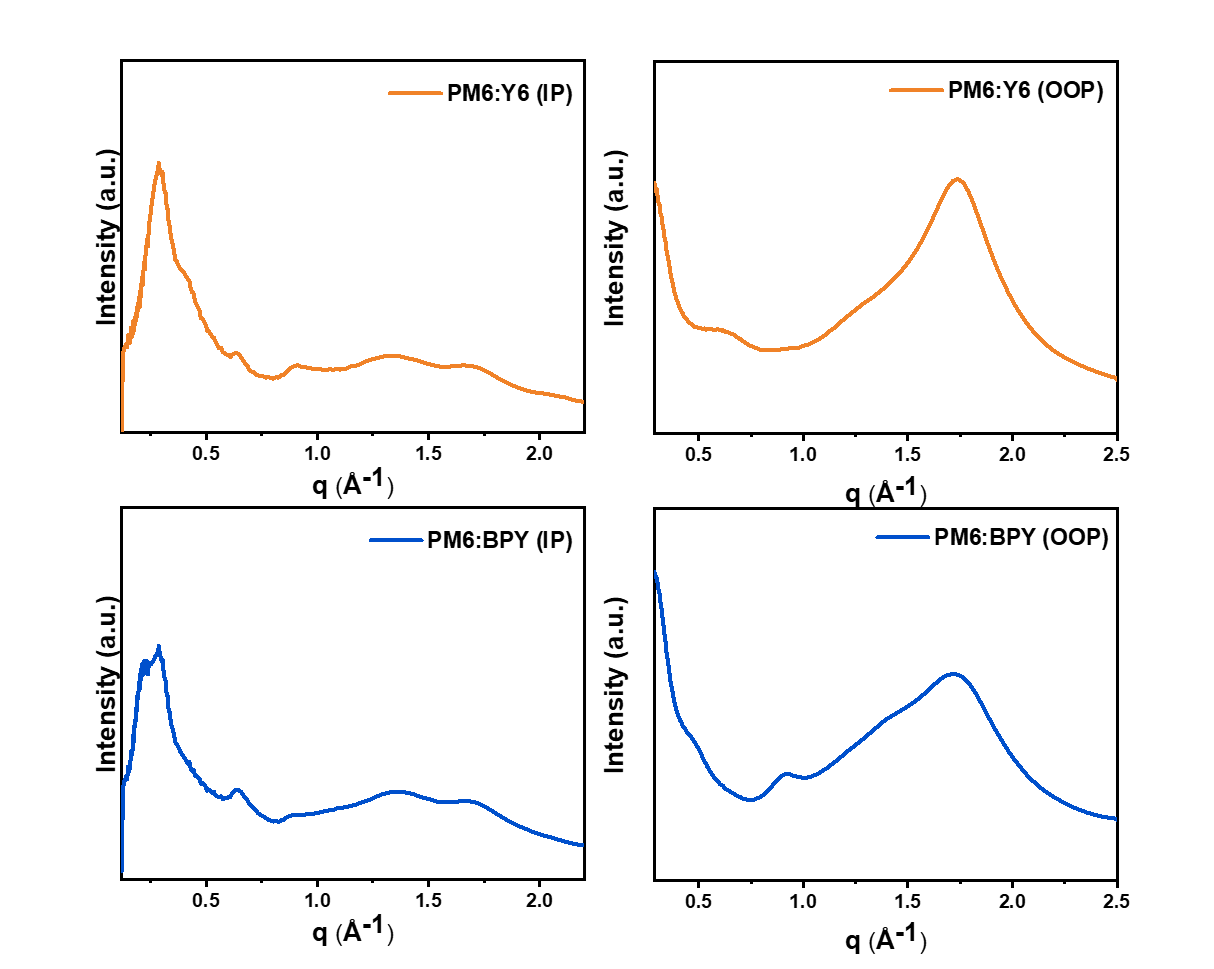


Figure S18. GIXD line-cut images of PM6:Y6 and PM6:BPY blend films.

**Table S3.** Crystallographic parameters of PM6:Y6 and PM6:BPY blend films.


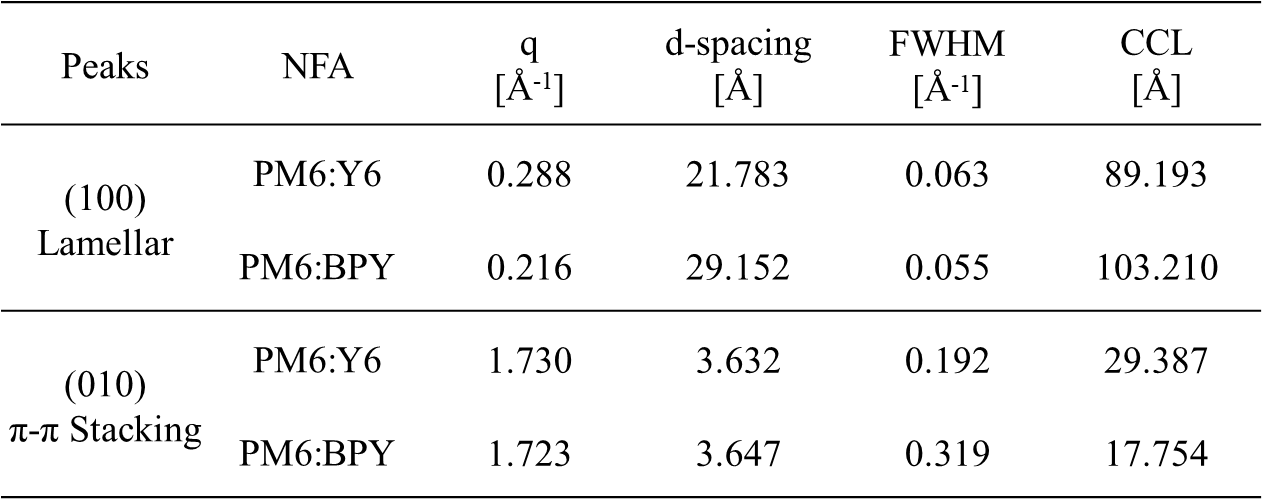


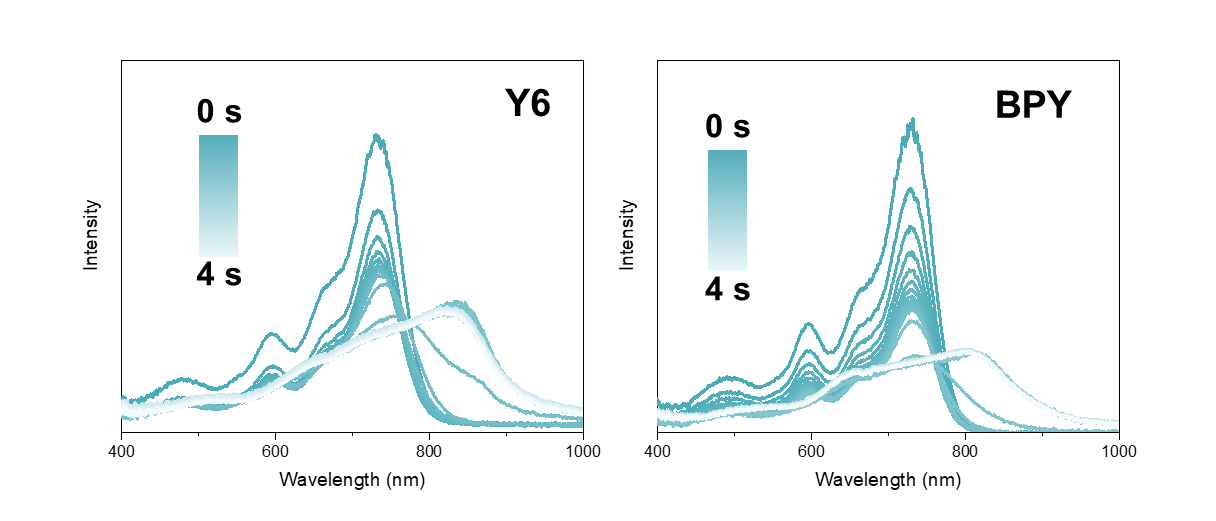


Figure S19. The evolution spectra of the PL spectra of Y6 and BPY.


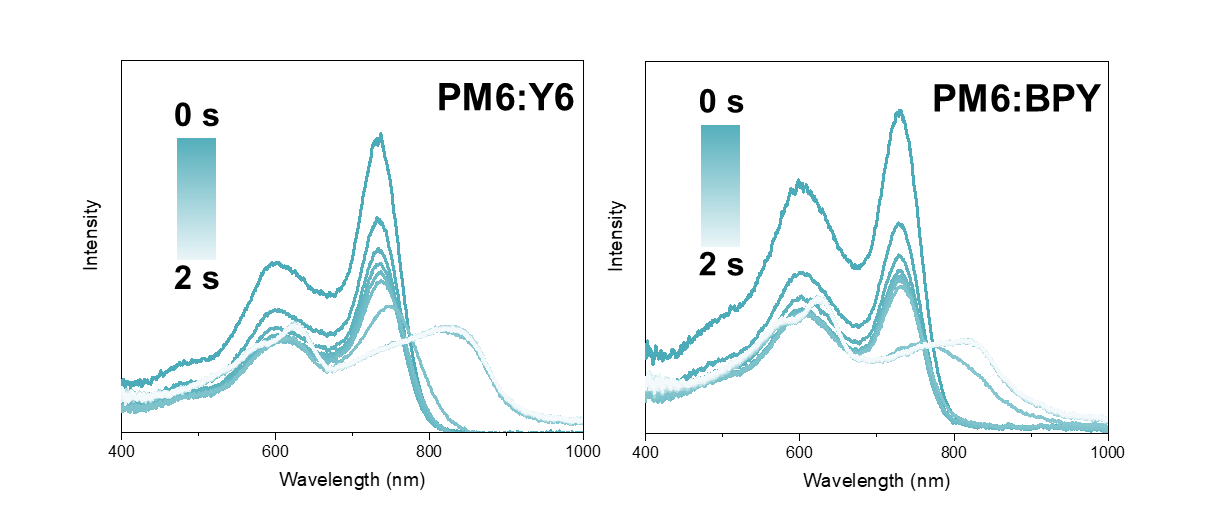


Figure S20. The evolution spectra of the PL spectra of PM6:Y6 and PM6:BPY.

Figure S21. ^1^H NMR spectrum of compound 2.

Figure S22. ^1^H NMR spectrum of compound 4.

Figure S23. ^1^H NMR spectrum of compound 6.


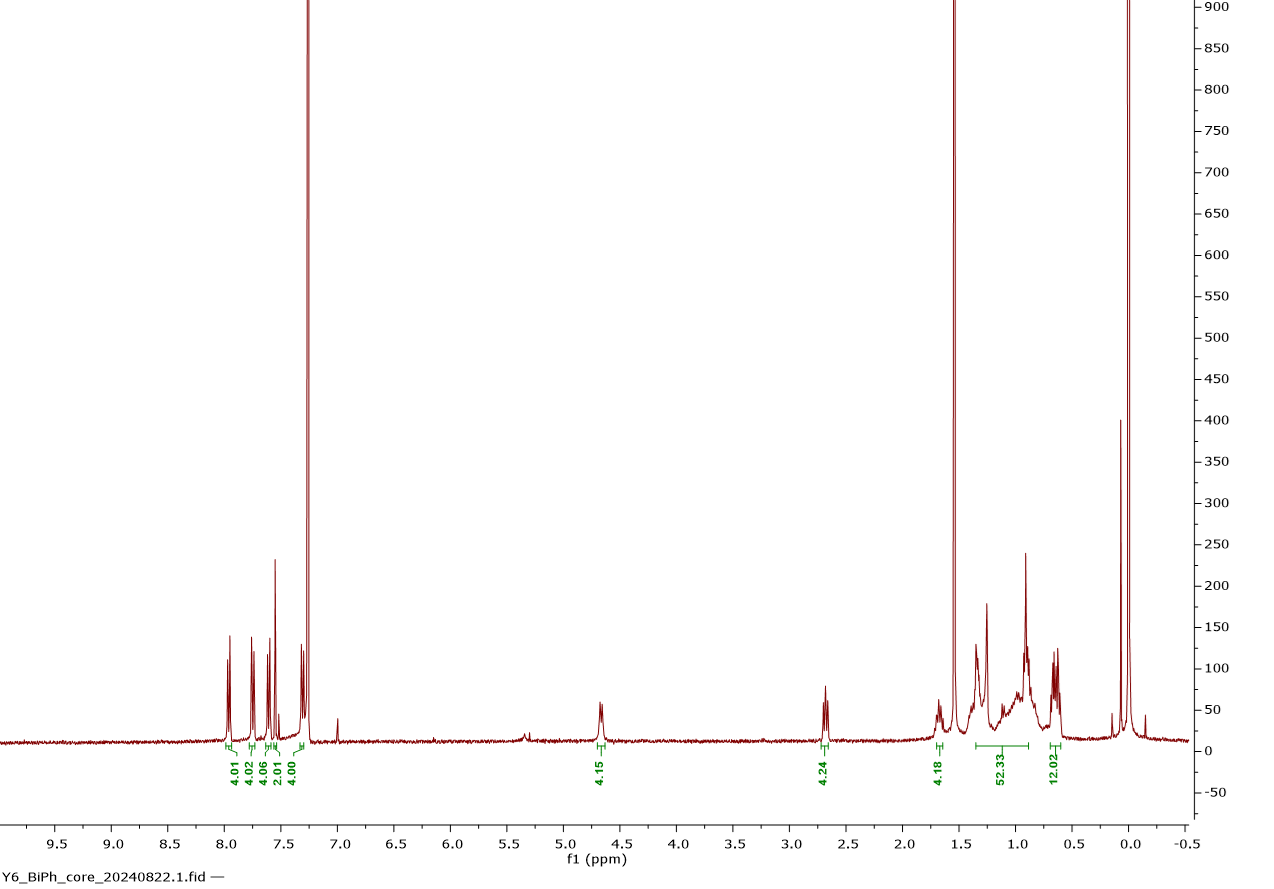


Figure S24. ^1^H NMR spectrum of compound 7.


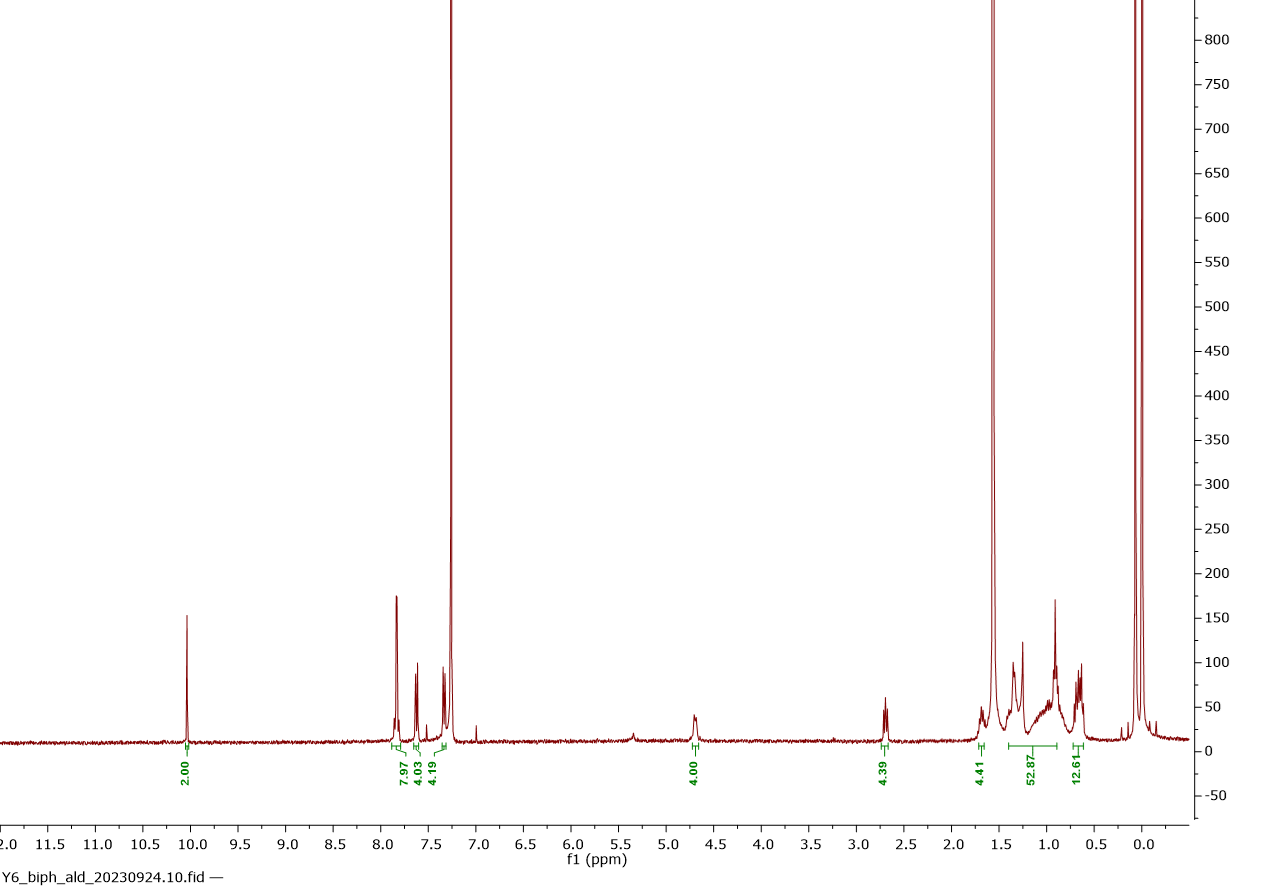


Figure S25. ^1^H NMR spectrum of compound 8.


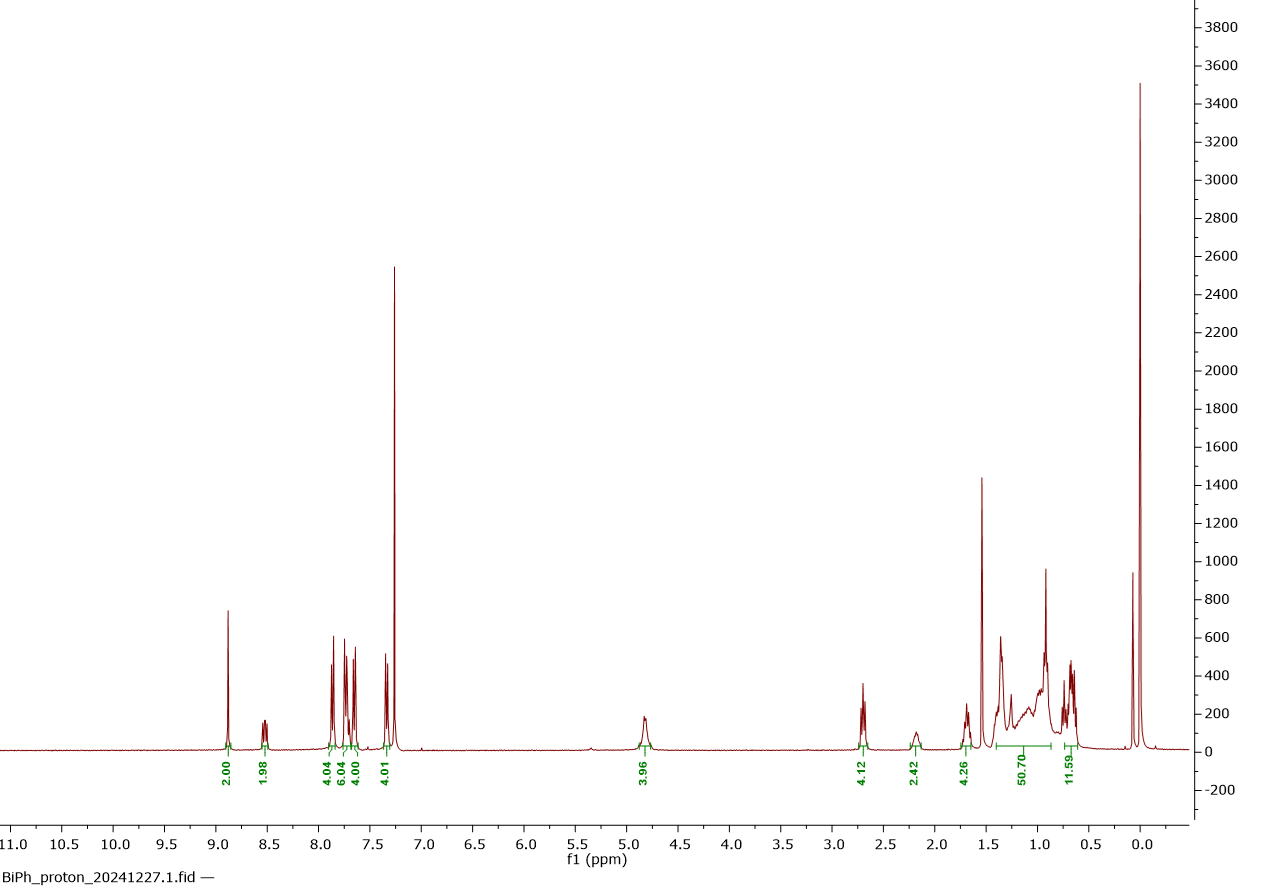


Figure S26. ^1^H NMR spectrum of BPY.

Figure S27. ^13^C NMR spectrum of BPY.
